# Multi-omic profiling of plasma reveals molecular alterations in children with COVID-19

**DOI:** 10.7150/thno.61832

**Published:** 2021-07-06

**Authors:** Chong Wang, Xufang Li, Wanshan Ning, Sitang Gong, Fengxia Yang, Chunxiao Fang, Yu Gong, Di Wu, Muhan Huang, Yujie Gou, Shanshan Fu, Yujie Ren, Ruyi Yang, Yang Qiu, Yu Xue, Yi Xu, Xi Zhou

**Affiliations:** 1 Guangzhou Institute of Pediatrics, Guangzhou Women and Children's Medical Center, Guangzhou Medical University, Guangzhou, 510120, China; 2 Department of Infectious Diseases, Guangzhou Women and Childrens Medical Center, Guangzhou, 510120, China; 3 State Key Laboratory of Virology, Wuhan Institute of Virology, Center for Biosafety Mega-Science, Chinese Academy Sciences, Wuhan, Hubei 430071, China; 4 MOE Key Laboratory of Molecular Biophysics, Hubei Bioinformatics and Molecular Imaging Key Laboratory, Center for Artificial Intelligence Biology, College of Life Science and Technology, Huazhong University of Science and Technology, Wuhan, Hubei 430074, China

## Abstract

**Rationale:** Children usually develop less severe symptoms responding to Coronavirus Disease 2019 (COVID-19) than adults. However, little is known about the molecular alterations and pathogenesis of COVID-19 in children.

**Methods:** We conducted plasma proteomic and metabolomic profilings of the blood samples of a cohort containing 18 COVID-19-children with mild symptoms and 12 healthy children, which were enrolled from hospital admissions and outpatients, respectively. Statistical analyses were performed to identify molecules specifically altered in COVID-19-children. We also developed a machine learning-based pipeline named inference of biomolecular combinations with minimal bias (iBM) to prioritize proteins and metabolites strongly altered in COVID-19-children, and experimentally validated the predictions.

**Results:** By comparing to the multi-omic data in adults, we identified 44 proteins and 249 metabolites differentially altered in COVID-19-children against healthy children or COVID-19-adults. Further analyses demonstrated that both deteriorative immune response/inflammation processes and protective antioxidant or anti-inflammatory processes were markedly induced in COVID-19-children. Using iBM, we prioritized two combinations that contained 5 proteins and 5 metabolites, respectively, each exhibiting a total area under curve (AUC) value of 100% to accurately distinguish COVID-19-children from healthy children or COVID-19-adults. Further experiments validated that all the 5 proteins were up-regulated upon coronavirus infection. Interestingly, we found that the prioritized metabolites inhibited the expression of pro-inflammatory factors, and two of them, methylmalonic acid (MMA) and mannitol, also suppressed coronaviral replication, implying a protective role of these metabolites in COVID-19-children.

**Conclusion:** The finding of a strong antagonism of deteriorative and protective effects provided new insights on the mechanism and pathogenesis of COVID-19 in children that mostly underwent mild symptoms. The identified metabolites strongly altered in COVID-19-children could serve as potential therapeutic agents of COVID-19.

## Introduction

The pandemic of Coronavirus Disease 2019 (COVID-19) caused by severe acute respiratory syndrome coronavirus 2 (SARS-CoV-2) has become the worst public health crisis once a century, which has caused almost 180 million infections and 4 million deaths all over the world as of June 23, 2021. It has been found that all people are susceptible to SARS-CoV-2 without significant differences in sex or age 1-3, and SARS-CoV-2 infects children under 18-year-old at a similar rate as adults 4. Reports from different countries showed that the symptoms are milder in the overwhelming majority of COVID-19-children compared to that of COVID-19-adults 1-3, 5-10. Most COVID-19-children have minor symptoms or asymptomatic infections, whereas severe conditions such as acute respiratory distress syndrome and multisystem inflammatory syndrome are rare in COVID-19-children 11-13.

Several theories have been proposed to explain the differences in clinical symptoms between children and adults infected with COVID-19 14. One plausible theory is that children might have a distinct response to SARS-CoV-2 in comparison with adults, mainly attributed to the differences in the composition and functional responsiveness of the immune system between children and adults 15, 16. For example, T and B cell responses to novel pathogens in children, such as natural antibodies rapidly produced from memory B cells, were not seen in adults 17. Also, young children are usually infected with other simultaneous viruses in the mucosa of lungs and airways 18, which might restrict the infection of SARS-CoV-2 via virus to virus interaction and competition. In addition, differences in the maturity and function of the viral entry receptor angiotensin-converting enzyme (ACE2) between children and adults might differentially influence the cellular entry of SARS-CoV-2 19. So far, the molecular alterations in COVID-19-children remain to be studied, and such an effort will be helpful for better understanding the detailed mechanisms underlying the differences in clinical symptoms between children and adults.

Recently, multiple reports showed that the immune system of COVID-19-children is less likely to elicit an excessive inflammatory response and cytokine storm, as frequently observed in COVID-19-adults 19-22. Thus, one explanation is that global molecular alterations in COVID-19-children might be milder, and the deteriorative process of COVID-19 might not be strongly induced in COVID-19-children. However, children are more susceptible to other infections 17, and molecular alterations in COVID-19-children might also be dramatically induced, whereas some protective mechanisms might be elicited to antagonize the deterioration of the disease. To test this hypothesis, we collected plasma samples from a Chinese cohort of 18 COVID-19-children and 12 healthy children, and conducted both proteomic and metabolomic profilings. We compared the multi-omic data of children to those of adults with or without COVID-19 that were quantified in our previous studies 23, 24, and uncovered numerous molecular alterations specifically occurred in COVID-19-children against healthy children or COVID-19-adults. Moreover, we developed a new pipeline named inference of biomolecular combinations with minimal bias (iBM), and predicted 5 proteins and 5 metabolites to be strongly altered in COVID-19-children. Further analyses revealed an antagonistic effect that both deteriorative immune response/inflammation processes and protective antioxidant or anti-inflammatory processes were up-regulated to a strong extent, indicating that the immune system of COVID-19-children might be in a relatively balanced state to both restrict SARS-CoV-2 infection and prevent the deterioration of the disease. Following experiments not only validated the changes of the 5 proteins in expression upon coronavirus infection, but also uncovered the regulatory roles of the prioritized metabolites in suppressing viral replication and inflammation. Taken together, our findings shed lights on a better understanding of the mild COVID-19 symptom in children, and provided candidate therapeutic agents for further treatment of the disease.

## Results

### Study design and blood samples

**Figure 1:**
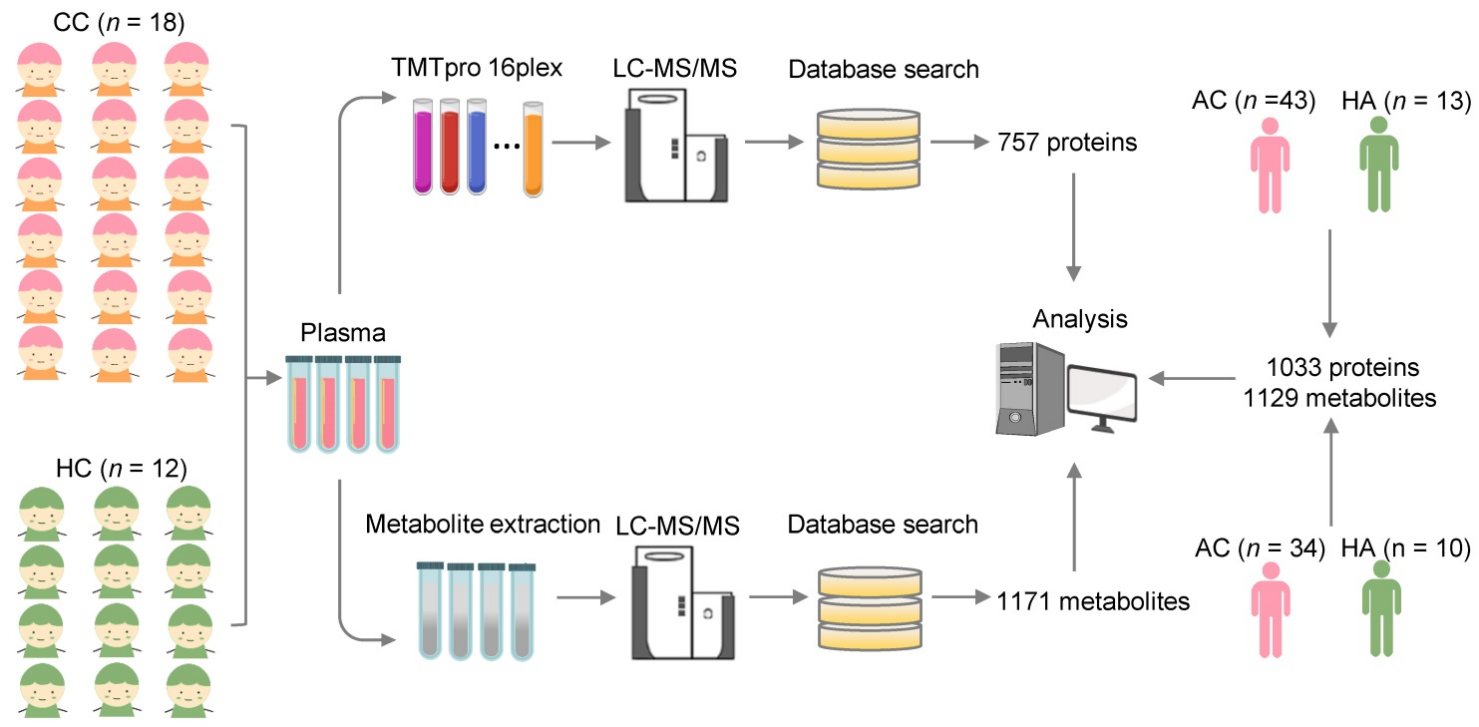
** Study design and blood samples.** Overview of plasma samples collected from COVID-19-children (*n* = 18) and healthy children (*n* = 12). The workflow for processing the proteomic and metabolomic data was shown, including the plasma separation, TMTpro 16-plex labeling, metabolite extraction, LC-MS/MS analysis, database search and further computational analyses. The proteomic data of 43 COVID-19-adults and 13 healthy adults, and metabolomic data of 34 COVID-19-adults and 10 healthy adults were taken from our previous studies 23, 24, and used for further computational analyses. CC, COVID-19-children; HC, healthy children; AC, COVID-19-adults; HA, healthy adults.

We collected the blood samples of 30 children including 18 COVID-19-children and 12 healthy children from Guangzhou Women and Children's Medical Center (Figure 1). All COVID-19-children were diagnosed as mild symptoms based on the Diagnosis and Treatment Protocol for Novel Coronavirus Pneumonia (6^th^ edition) of the National Health Commission of China 25, and discharged from the hospital after recovery. The clinical data of the 18 COVID-19-children was shown, and no severe or critically ill cases were charged in our cohort (Table 1). The 12 healthy children, whose throat swab tests and serological tests were negative for SARS-CoV-2, were enrolled for comparison (Table 1).

For each blood sample, plasma was separated. For the proteomic profiling, the 30 plasma samples containing digested peptides were separated into 2 batches, subjected to tandem mass tag (TMT) labeling (Table S1), and analyzed by liquid chromatography with tandem mass spectrometry (LC-MS/MS), which was also used for the metabolomic quantification. In total, we obtained 757 proteins and 1171 metabolites quantified in at least one sample (Table S2, S3). More details on the quality control and analysis of the multi-omic data were present in Supplementary results. Recently, we also conducted metabolomic and proteomic profilings of plasma samples from adults with or without SARS-CoV-2 infection, and identified numerous molecular alterations associated with COVID-19 in adults 23, 24. From our two previous studies, we obtained the TMT-based quantitative proteomic data of 43 COVID-19-adults and 13 healthy adults, and metabolomic data of 34 COVID-19-adults and 10 healthy adults. In total, there were 1033 proteins and 1129 metabolites quantified in at least one sample of COVID-19-adults and healthy adults.

### Characterization of molecular alterations in COVID-19-children

**Figure 2:**
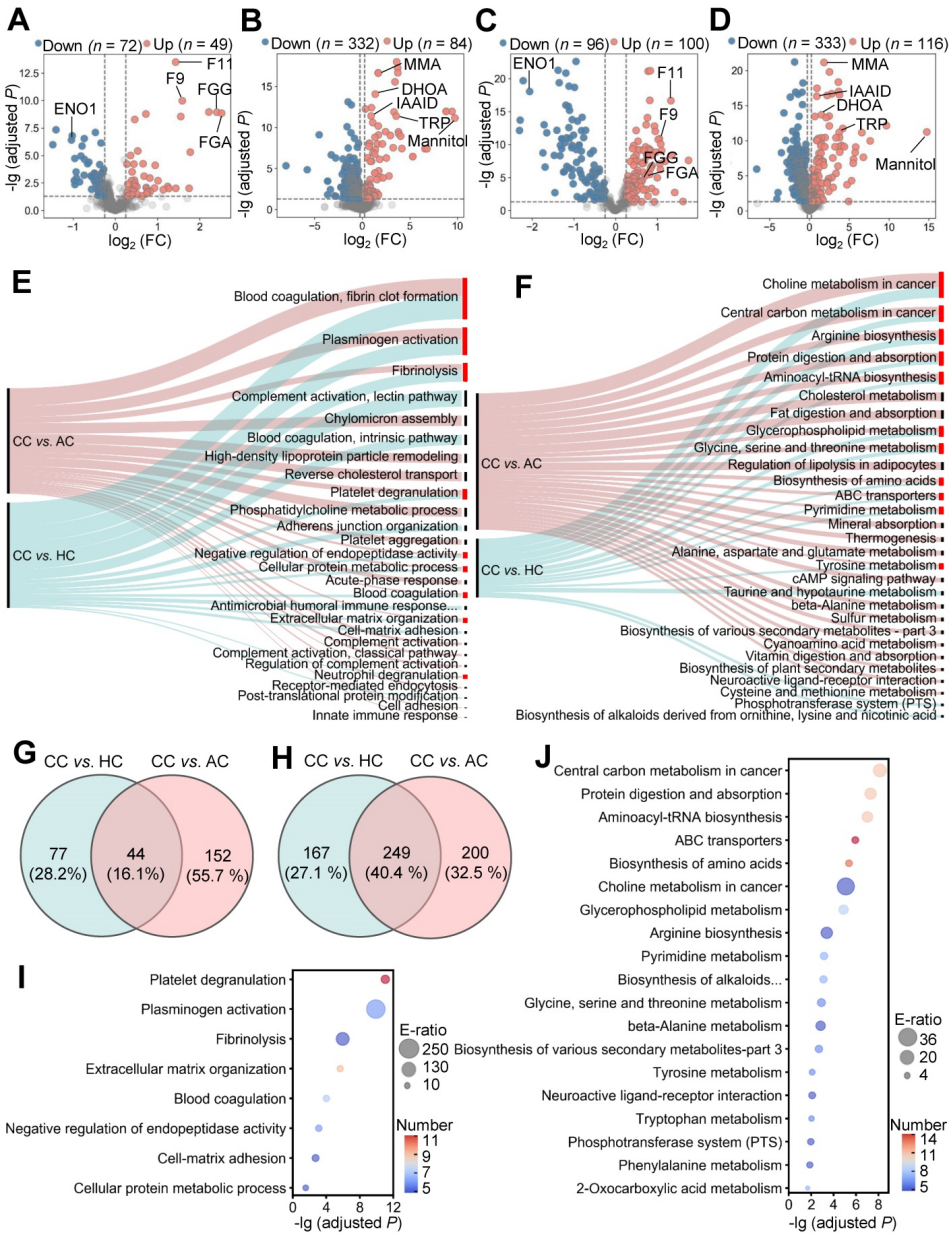
**Proteomic and metabolomic alterations specifically occurred in COVID-19-children. A,B** Volcano plots show the protein (A) and metabolite (B) alterations in COVID-19-children against healthy children. **C,D** Volcano plots show the protein (C) and metabolite (D) alterations in COVID-19-children against COVID-19-adults. Proteins and metabolites with |log_2_(FC)| > 0.25 with an adjusted *P* < 0.05 were considered as potential DEPs and DEMs, respectively. **E** GO-based enrichment analysis for DEPs of COVID-19-children against healthy children or COVID-19-adults (Two-sided hypergeometric test, *m* ≥ 5, adjusted *P* < 10^-5^). **F** KEGG-based enrichment analysis for DEMs of COVID-19-children against healthy children or COVID-19-adults (Two-sided hypergeometric test, *m* ≥ 5, adjusted *P* < 10^-3^). **G,H** DEPs (G) and DEMs (H) specifically altered in COVID-19-children were identified by overlapping DEPs and DEMs of COVID-19-children against healthy children and against COVID-19-adults, respectively.** I** GO-based enrichment analysis of DEPs specifically altered in COVID-19-children shown in the term of biological processes (Two-sided hypergeometric test, *m* ≥ 5, adjusted *P* < 0.01).** J** KEGG-based enrichment analysis of DEMs specifically altered in COVID-19-children (Two-sided hypergeometric test, *m* ≥ 5, adjusted *P* < 0.01). CC, COVID-19-children; HC, healthy children; AC, COVID-19-adults.

To identify molecular alterations in COVID-19-children against healthy children, we used relative protein abundances (RPAs) of the proteomic data and intensity-based abundances (IBAs) of the metabolomic data, and in total detected 121 differentially expressed proteins (DEPs) and 416 differentially expressed metabolites (DEMs), respectively (Figure 2A-B, |log_2_(fold-change or FC)| > 0.25, Adjusted *P* < 0.05). It could be found that more DEPs and DEMs were down-regulated in COVID-19-children, indicating a generally suppressive effect of normal biological processes in children upon SARS-CoV-2 infection (Figure 2A-B and Table S4-S5). However, up-regulated molecules exhibited stronger changes in expression, supporting that COVID-19-associated molecular alterations are also induced in children.

To further identify molecular alterations in COVID-19-children against COVID-19-adults, the intrinsic differences between adults and children were eliminated by calculating normalized abundance values (NAVs) of proteins and metabolites in COVID-19-children or COVID-19-adults against their counterparts in the samples of healthy children or adults, respectively. Again, 332 proteins and 783 metabolites simultaneously quantified in > 80% children and adults were reserved to ensure the data quality, and principal component analysis (PCA) demonstrated that COVID-19-children and COVID-19-adults can be clearly distinguished either by the proteomic or metabolomic data (Figure S4A-B). Using NAVs, we identified 196 DEPs and 449 DEMs in COVID-19-children against COVID-19-adults (Figure 2C-D, |log_2_(FC)| > 0.25, Adjusted *P* < 0.05, Table S6-S7). Although the numbers of up- and down-regulated DEPs were similar in COVID-19-children, much more metabolites were down-regulated in COVID-19-children, indicating a stronger suppressive effect of metabolic process in COVID-19-children than in COVID-19-adults.

Next, we used the annotations of Gene Ontology (GO) biological processes and Kyoto Encyclopedia of Genes and Genomes (KEGG) pathways, and performed functional enrichment analyses for proteins and metabolites, respectively (Figure 2E-F and Table S8-S9). We found that a considerable number of biological processes and metabolic pathways enriched in COVID-19-children against COVID-19-adults or healthy children were overlapped, such as platelet degranulation (GO:0002576), blood coagulation (GO:0007596), fibrinolysis (GO:0042730) and plasminogen activation (GO:0031639) in the proteomic level (Figure 2E), and ABC transporters (KEGG ID: map02010), biosynthesis of amino acids (KEGG ID: map01230) and pyrimidine metabolism (KEGG ID: map00240) in the metabolic level (Figure 2F). Although most of these processes/pathways were also enriched in the blood samples of COVID-19-adults 23, 24, they were altered to a much stronger extent in COVID-19-children. By overlapping DEPs and DEMs of COVID-19-children against healthy children or COVID-19-adults, we identified 44 DEPs and 249 DEMs specifically altered in COVID-19-children, respectively (Figure 2G-H and Table S10-S11). We performed functional enrichment analyses for these DEPs (Figure 2I) and DEMs (Figure 2J and Table S8-S9), respectively. Again, blood coagulation-related processes were highly enriched at the proteomic level, while anabolism-related pathways involved in amino acid biosynthesis were enriched in the metabolic level (Figure S4C-D), suggesting potential roles of these physiological changes in children responding to COVID-19.

### Machine learning-based inference of molecules strongly altered in COVID-19-children

**Figure 3:**
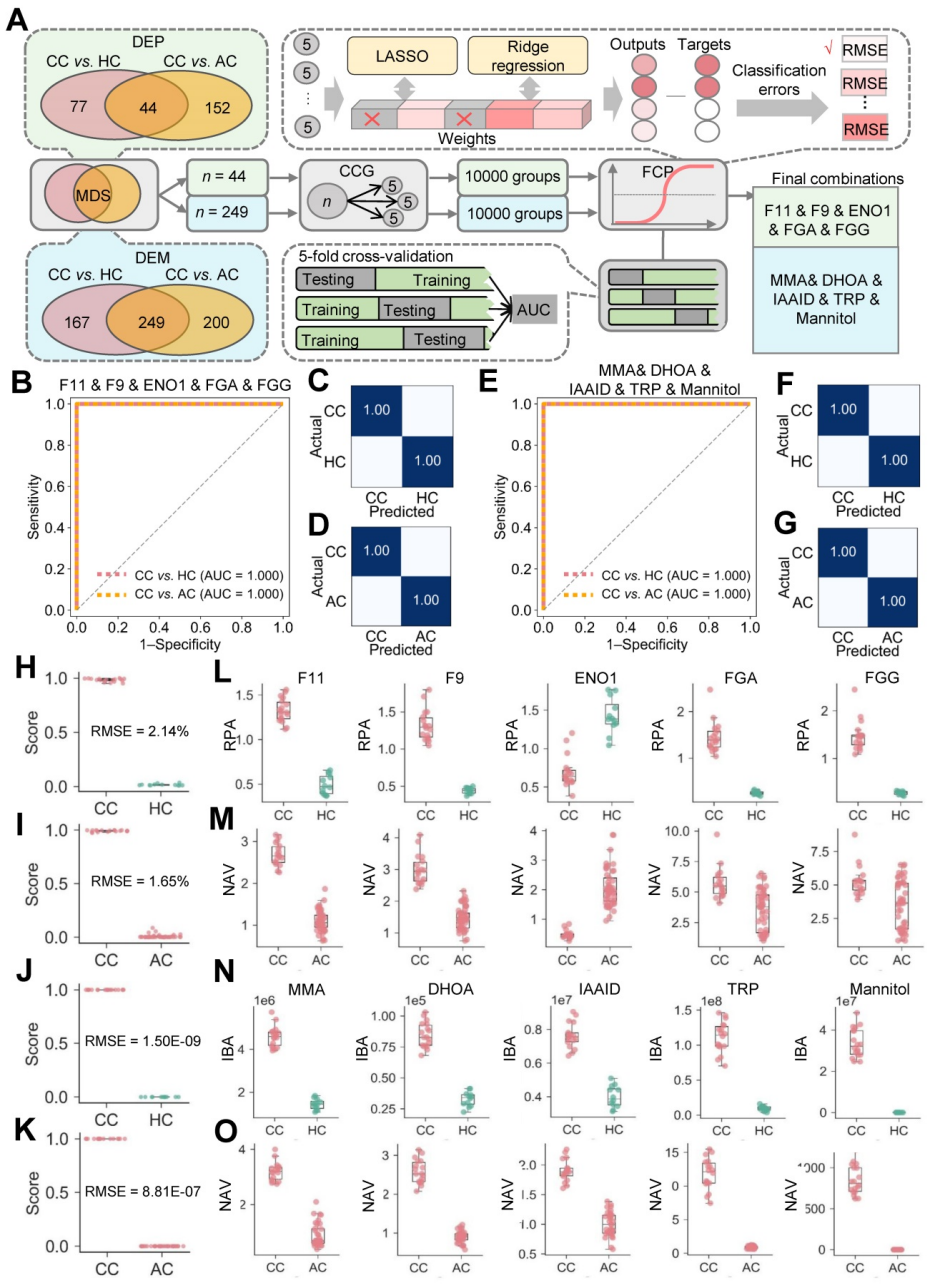
** Inference of biomolecular combinations specifically altered in COVID-19-children using a machine learning strategy. A** The workflow of iBM, including MDS, CCG and FCP to prioritize candidate combinations with a maximal accuracy and a minimal bias from the 5-fold cross-validation. **B** From the 5-fold cross-validation, AUC values of the protein combination for distinguishing COVID-19-children from healthy children or COVID-19-adults was calculated. **C,D** The confusion matrices of the protein combination for distinguishing COVID-19-children from healthy children (C) or COVID-19-adults (D). **E** From the 5-fold cross-validation, AUC values of the metabolite combination for distinguishing COVID-19-children from healthy children or COVID-19-adults was calculated.**F,G** The confusion matrices of the metabolite combination for distinguishing COVID-19-children from healthy children (F) or COVID-19-adults (G). **H,I** The RMSE results of the protein combination for distinguishing COVID-19-children from healthy children (H) or COVID-19-adults (I). **J,K** The RMSE results of the metabolite combination for distinguishing COVID-19-children from healthy children (J) or COVID-19-adults (K). **L,M** The expression levels of 5 proteins in COVID-19-children against healthy children (L) or against COVID-19-adults (M). **N,O** The expression levels of 5 metabolites in COVID-19-children against healthy children (N) or against COVID-19-adults (O). The center line within each box showed the median, and the top and bottom of each box represented the 75^th^ and 25^th^ percentile values, respectively. The upper and lower whiskers extended from the hinge to the largest and smallest value no further than 1.5 times the distance between the first and third quartiles, respectively. CC, COVID-19-children; HC, healthy children; AC, COVID-19-adults.

Although 44 DEPs and 249 DEMs were identified (Figure 2G-H), different molecules were altered with distinct extents in COVID-19-children. Identification of optimal biomolecular combinations will be not only helpful for accurate classification of different types of patients, but also provide useful information for uncovering the potential pathogenesis of COVID-19 in children. Here, we developed a new pipeline named iBM, which consisted of three steps, including mutual DEPs or DEMs selection (MDS), candidate combination generation (CCG) to randomly select 10,000 combinations, and final combination prioritization (FCP) to get the protein or metabolite combination with a maximal accuracy and a minimal bias through the 5-fold cross-validation (Figure 3A). The accuracy of a candidate model was evaluated by calculating the total area under curve (AUC) value, and we also computed the total root mean squared error (RMSE) to measure the prediction bias. In the step of FCP, a widely-used machine learning algorithm, penalized logistic regression (PLR) 26-28, was used for model training and parameter optimization (Figure 3A). The combinations were separately determined for the proteomic and metabolic data.

From the results, there were 8098 protein combinations and 8376 metabolite combinations with a total AUC value of 1 (Table S12-S13), indicating that too many combinations could achieve a perfect accuracy on the existing data. However, a minimal RMSE value between predicted scores and observed values will ensure the robustness and reliability of the model on the new data. With total RMSE values of 1.83% and 7.01E-07, we prioritized two optimal combinations, containing 5 proteins coagulation factor XI and IX (F11 and F9), enolase (ENO1), fibrinogen alpha (FGA) and gamma (FGG) chains, and 5 metabolites methylmalonic acid (MMA), dihydroorotic acid (DHOA), indoleacetaldehyde (IAAld), tryptophan (TRP) and mannitol (Figure 3A). Both of the two combinations could perfectly distinguish COVID-19-children from healthy children or COVID-19-adults, with an AUC value of 1 (Figure 3B-G). Moreover, the results of confusion matrices and RMSE analyses of these combinations also showed a high accuracy for classifying different samples (Figure 3C, D, F, G and H-O).

Also, we calculated the total AUC values and total RMSE values for individual proteins or metabolites. For the 5 proteins, the total AUC values ranged from 0.77 to 1, and the total RMSE values ranged from 6.57% to 36.13% (Figure S5A-E, Table S14). For the 5 metabolites, all the total AUC values were 1, while the total RMSE values ranged from 10.97% to 28.72% (Figure S5F-J, Table S14). Although individual molecules can reach a perfect accuracy on the current data, the combination of multiple molecules was undoubtedly important to reduce the prediction bias.

### The molecular alterations strongly induced in COVID-19-children were linked with the mild clinical symptom

Besides the classification of different samples, the prioritized proteins and metabolites as well as other molecules specifically altered in COVID-19-children could also partially explain the differences in clinical symptoms between COVID-19-children and COVID-19-adults. In the protein combination, 4 proteins including F11, F9, FGA and FGG are involved in the blood coagulation cascade, and all of them were higher expressed in COVID-19-children than those in healthy children or COVID-19-adults (Figure 3L-M, Table S10). F11 and F9 are involved in the initiation of the thrombin generation during blood coagulation by proteolytic activation of a serial of coagulation factors 29. FGA and FGG contribute to form the fibrin clot in response to explosive generation of thrombin mediated by coagulation factors 30. Also, a Ca^2+^ binding protein S100A9, which induces inflammatory cytokine secretion and immune cell migration during inflammation 31, was higher expressed COVID-19-children (Table S10). These results indicated that immune response/inflammation processes were significantly induced in COVID-19-children. On the contrary, we found a number of plasma serine protease inhibitors such as SERPINA5, SERPINC1, and SERPINF2, which negatively regulate the blood coagulation cascade 29, were strongly up-regulated in COVID-19-children against in healthy children or COVID-19-adults (Table S10). Interestingly, ENO1, a key enzyme in the last steps of the catabolic glycolytic pathway, was significantly downregulated in COVID-19-children (Figure 3L-M, Table S10). It was reported that ENO1 is required for hypoxia-induced metabolic reprogramming from mitochondrial respiration to glycolysis 32, which is important to enhance the oxidant stress and inflammation 33. Glycolysis is strongly induced upon SARS-CoV-2 infection in human colonic carcinoma Caco-2 cells, whereas blocking this pathway using its inhibitor 2-deoxy-D-glucose (2-DG) markedly reduced SARS-CoV-2 replication 34. Thus, our results indicated that anti-inflammatory processes were also strongly triggered in COVID-19-children.

In the metabolite combination, all the 5 metabolites were significantly up-regulated in COVID-19-children against in healthy children or COVID-19-adults (Figure 3N-O, Table S11). DHOA is involved in the pyrimidine metabolism, and the secretion of DHOA can reduce the toxicity of glucose metabolism reprogramming in response to hypoxia 35. TRP could be metabolized to other indole compounds such as IAAld. The downstream products of TRP such as N-formylkynurenine (KYN) and its intermediates, are agonists to activate the aryl hydrocarbon receptor (AhR), which contributes to the immunosuppression and restriction of inflammation 36. In particular, the expression of KYN in COVID-19-children was lower than in healthy children, but much higher than in COVID-19-adults (Table S11), suggesting that AhR was still active and the SARS-CoV-2-induced immune response/inflammation processes was still under the control in COVID-19-children. Furthermore, mannitol has been found to be a hydroxyl radical scavenger that plays important roles in reducing inflammation 37. In addition, MMA is a dicarboxylic acid that is primarily a by-product of the propionate metabolism. It was shown that the elevated circulating MMA level caused an up-regulated expression of Sex-determining region Y box 4 (SOX4), which consequently promoted transcriptional reprogramming 38. The role of MMA in the regulation of immune response and inflammation remained to be dissected. Besides the 5 prioritized metabolites, we also found other metabolites having the potential to relieve the exacerbated inflammation. For example, uric acid (UA), an end product of purine catabolism, is a major antioxidant in the blood, and can be helpful for protection against free-radical oxidative damage 39. The plasma level of UA in COVID-19-children was > 109-fold higher than that in COVID-19-adults (Table S11).

Together, our findings indicated that both deteriorative immune response/inflammation processes and protective antioxidant or anti-inflammatory processes were significantly elevated in the circulating system of COVID-19-children compared with healthy children or COVID-19-adults. Consistent with this, the clinical data of COVID-19-children showed that the levels of coagulation indicators such as APTT, PT and D-dimer, the status of immune cell activation such as the ratios of CD3^+^CD4^+^ and CD3^+^CD8^+^, and the levels of inflammatory factors such as IFN-γ and IL-1β, generally followed in the normal range (Table 1).

### Validation of expressions or regulatory roles of prioritized molecules upon coronavirus infection

**Figure 4:**
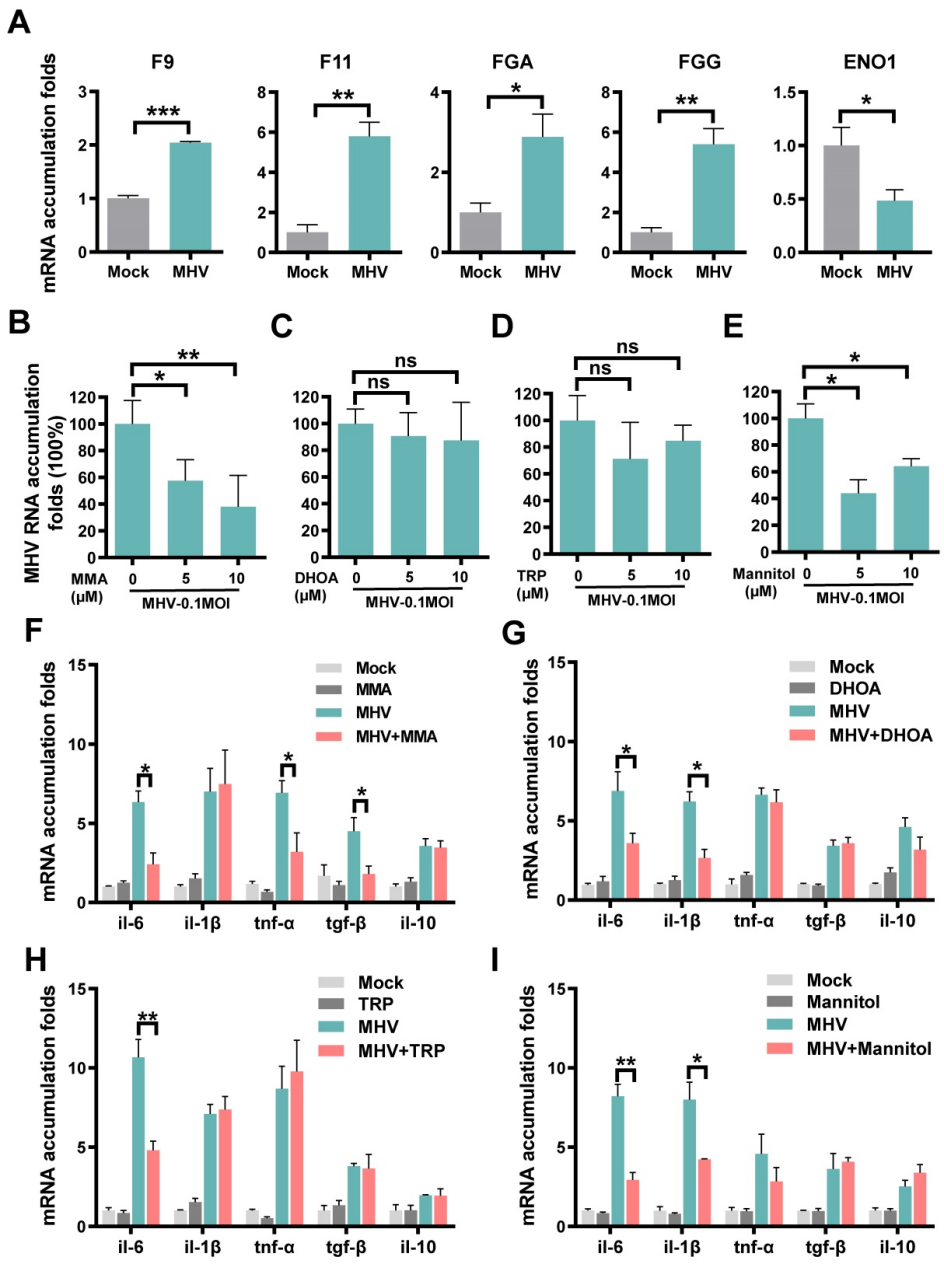
** Expressions or regulatory roles of molecules prioritized by iBM upon coronavirus infection. A** Rat lung epithelial L2 cells were infected with MHV at MOI of 0.1. At 12 h post-infection, total cellular RNAs were extracted, and the mRNA levels of F9, F11, FGA, FGG and ENO1 were examined using qRT-PCR before (Mock) and after infection. **B-E** L2 cells were treated with MMA (B), DHOA (C), TRP (D) or mannitol (E) at the concentration of 5 or 10 μM, respectively, for 1 h, and then infected with MHV at MOI = 0.1, respectively. At 12 h post-infection, the total cellular RNAs were extracted. The viral RNA accumulations were determined via qRT-PCR with or without treatment of each of the metabolites. **F-I** The mRNA levels of IL-6, IL-1β, TNF-α, TGF-β and IL-10 were determined via qRT-PCR before (Mock) and after infection, as well as with or without treatment of each of the metabolites. The qRT-PCR results were statistically analyzed by *t*-test (GraphPad Prism). ns > 0.05, *P < 0.05, **P < 0.05, and ***P < 0.001.

For the protein combination, we measured their mRNA expression changes in cells with or without coronavirus infection, using the quantitative reverse transcription PCR (qRT-PCR). We used the mouse hepatitis virus (MHV, strain A59) 40, a well-known surrogate for SARS-CoV-2 41, to infect rat lung epithelial L2 cells at a multiplicity of infection (MOI) of 0.1. At 12 h post-infection, total cellular RNAs were extracted and the mRNA expression of each protein was separately examined (Table S15). In consistent with the proteomic data, MHV infection resulted in significantly enhanced mRNA levels of F9, F11, FGA and FGG, and markedly reduced the mRNA expression of ENO1 (Figure 4A).

For the metabolite combination, we individually exploited their functional impacts on viral replication and inflammation in the context of MHV infection. IAAld was not probed because it's a downstream product of TRP and its biological impact is similar to TRP. For each of the remaining 4 metabolites, the cell counting kit-8 (CCK-8) assay was adopted to measure the 50% cytotoxic concentration (CC_50_) in L2 cells at different concentrations. The CC_50_ values of all the 4 metabolites were greater than 1280 μM, indicating low cytotoxicity of the metabolites (Figure S6A-D). Then, for or each of the 4 metabolites at a concentration of 5 or 10 μM, L2 cells were pre-treated for 1 h, and then infected with MHV at an MOI of 0.1. At 12 h post-infection, total cellular RNAs were extracted and the viral RNA accumulation as well as the mRNA levels of 5 inflammatory cytokines, such as IL-6, IL-1β, TNF-α, TGF-β and IL-10, were examined using qRT-PCR. From the results, we found that two metabolites, MMA and mannitol but not DHOA or TRP, markedly reduced the RNA accumulation level of MHV (Figure 4B-E). Also, we found that the 4 metabolites could reduce the mRNA expression of at least one inflammatory cytokine in MHV-infected cells (Figure 4F-I). For example, the pre-treatment of MMA for 1 h could markedly reduce the mRNA expression of IL-6, TNF-α, and TGF-β (Figure 4F), whereas both IL-6 and IL-1β were down-regulated by the treatment of either DHOA or mannitol (Figure 4G,I). The pre-treatment of TRP only down-regulated the mRNA expression of IL-6 (Figure 4H). Taken together, our experiments not only validated the proteins that might be truly altered upon SARS-CoV-2 infection, but also revealed a protective role of some of the prioritized metabolites in inhibiting viral replication and inflammatory cytokines.

## Discussion

The infection rate of SARS-CoV-2 to children is similar to that of adults, but the clinical symptom is much milder in most cases of COVID-19-children 1-10. Therefore, a better understanding of the mechanisms underlying the milder COVID-19 symptom in children is particularly important to uncover the pathogenesis of this disease. For this purpose, we conducted a multi-omic study to profile plasma proteomic and metabolomic alterations in COVID-19-children and healthy children, and identified molecule alternations specifically occurred in COVID-19-children by comparing with the multi-omic data of adults with or without COVID-19. By developing a new pipeline named iBM, we prioritized two optimal biomolecular combinations, each of them containing 5 proteins or 5 metabolites. Each combination could accurately distinguish the samples of COVID-19-children against healthy children or COVID-19-adults, with a total AUC of 1. Previously, Cotugno et al. developed a similar pipeline to predict the immunogenicity of influenza trivalent inactivated vaccine in children infected with human immunodeficiency virus (HIV) 42. Similar to MDS, the first step of iBM, the differentially expressed genes were selected based on the Wilcoxon Rank Sum Test, from 96 profiled genes. Also, multiple machine learning methods were used to further narrow down the number of candidates, and this step was similar to the CCG step. Finally, an ensemble machine learning algorithm, Adaptive Boosting or AdaBoost, was used to determine the final gene combination with the best performance, and this step was also similar to our FCP. In that study, the prediction bias of the model was not estimated. To validate the iBM-based predictions, we probed the mRNA expression changes of the 5 prioritized proteins upon coronavirus infection, and the results were consistent with the proteomic data. In addition, we found 2 metabolites, MMA and mannitol, involved in suppressing viral replication, and 4 metabolites, MMA, DHOA, TRP and mannitol, to be functional in reducing inflammatory effect.

Compared to healthy children, the 5 metabolites were highly up-regulated in COVID-19-children, with FC values of 2.58 to DHOA, 11.78 to TPR, 1.90 to IAAld, 446.26 to mannitol, and 3.20 to MMA, respectively (Table S5, adjusted *P* < 0.05). All the 5 prioritized metabolites could be taken from the diet 43-47. DHOA is a derivative of vitamin B_13_ or orotic acid that widely exists in bovine milk and dairy products 45, whereas TRP is highly expressed in meats 46. Mannitol plays a critical role in energy reserves and osmoregulation, and is commonly found in vegetables such as celery and leek 43. The elevated levels of MMA and homocysteine in the serum are indicators of vitamin B_12_ deficiency 44, 47. However, no children underwent a special diet or vitamin B_12_ deficiency in our cohort, and homocysteine was not differentially expressed in COVID-19-children against healthy children in our plasma metabolomic data (Table S3, S5). Thus, the significant changes of the 5 metabolites in COVID-19-children might be more likely attributed to SARS-CoV-2 infection but not the diet.

**Figure 5:**
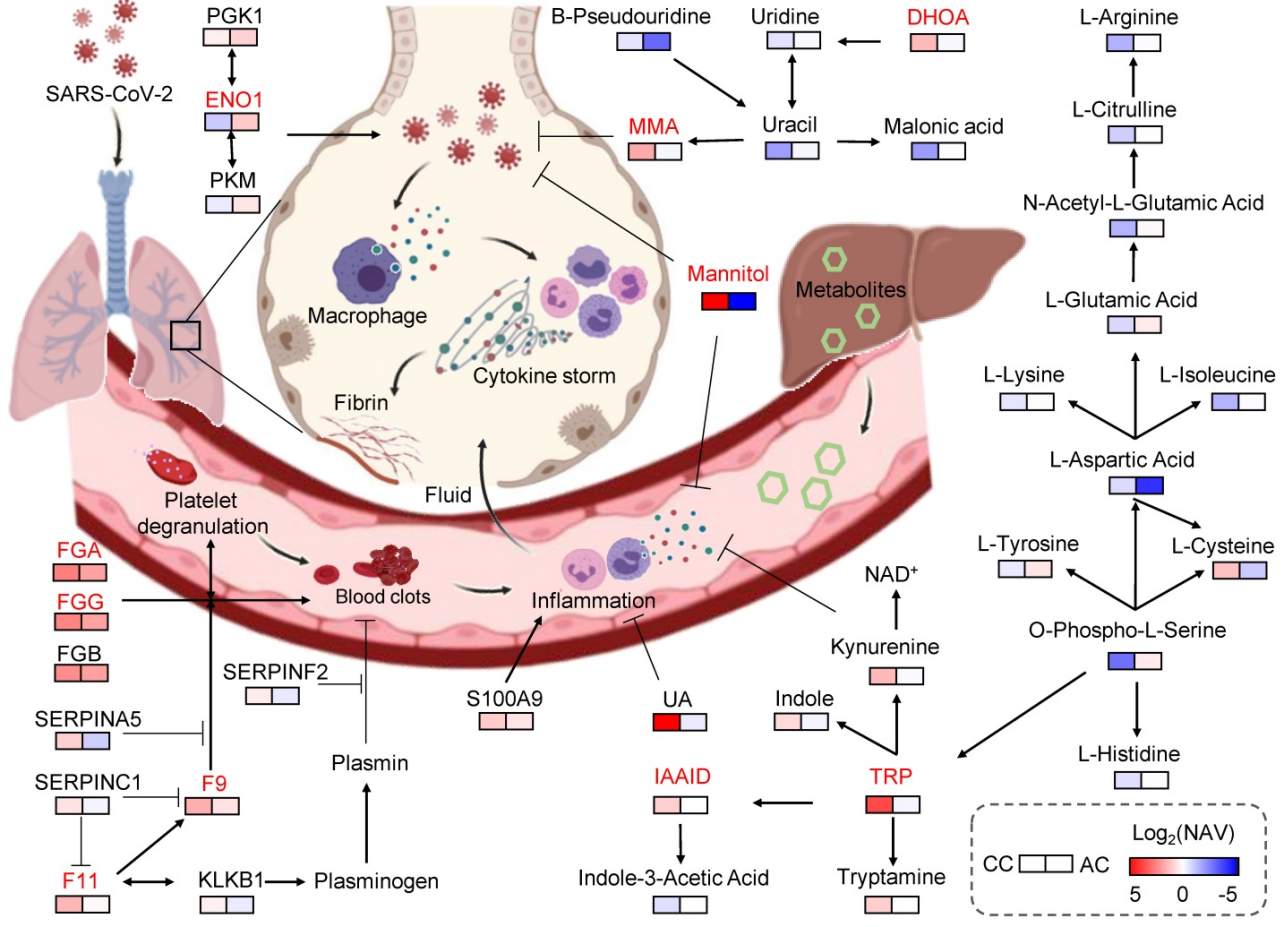
** Key proteins and metabolites specifically altered in COVID-19-children.** In this model, the plasma proteins involved in coagulation cascade were significantly higher expressed in COVID-19-children compared with COVID-19-adults, suggesting COVID-19-associated coagulation and the accompanying immune response/inflammation in COVID-19-children may be strongly active. On the other hand, the levels of many negative regulators of inflammation and oxidation, such as MMA, TRP, IAAld, DHOA, mannitol and UA in COVID-19-children were also significantly up-regulated compared with those in COVID-19-adults, indicating an antagonistic effect. Thus, the immune system in COVID-19-children is in a relatively balanced state, in which its activation is stronger than that of COVID-19-adults and is sufficient to restrict SARS-CoV-2 infection, as well as the collateral damages. Meanwhile, the molecules involved in anti-oxidant and anti-inflammatory processes were also strongly activated in COVID-19-children, thereby preventing the exacerbation of inflammation and the deterioration of disease. CC, COVID-19-children; AC, COVID-19-adults.

Then, we constructed a working model, using the 5 proteins and 5 metabolites prioritized by iBM, as well as other related molecules (Figure 5). Based on the pathway annotations in KEGG, this model included 13 DEPs and 25 DEMs strongly altered in COVID-19-children, and was mainly involved in 6 pathways, including platelet activation, complement and coagulation cascades, glycolysis/gluconeogenesis, pyrimidine metabolism, biosynthesis of amino acids and TRP metabolism. The homeostasis of all the 6 pathways is related to aging. In elders, the platelet reactivity and activation 48, the levels of pyrimidine intermediates 49, and biosynthesis of amino acids 50 are decreased, whereas the expressions of coagulation factors 51, the plasma glucose level 52, and the degradation of tryptophan 53 are enhanced. Thus, the multi-omic data of COVID-19-children and COVID-19-adults were normalized by using healthy children and healthy adults, respectively, before the comparative analysis. Such a procedure efficiently eliminated the intrinsic differences including age between adults and children (Figure S4A-B), and enabled an unbiased identification of DEPs and DEMs in COVID-19-children against COVID-19-adults (Table S6, S7).

In this study, the identified alternations of plasma proteins and metabolites as well as their enriched processes/pathways in COVID-19-children were largely in line with the previous omic profilings 22-24, 54-67. Previously, Shen et al. conducted the plasma proteomic and metabolomic profilings using a Chinese cohort of 46 COVID-19-adults and 53 healthy adults or non-COVID-19 patients, and identified 22 proteins and 7 metabolites as potential biomarkers of COVID-19-adults 54. Also, Messner et al. performed a plasma proteomic profiling with a German cohort containing 31 patients with different COVID-19 severity, and validated 27 severity-related DEPs 58. Based a Korean COVID-19 cohort containing 3 mild and 5 severe cases, Park et al. conducted a plasma proteomic profiling, and identified 76 DEPs to be potentially associated with the disease severity 59. The three studies identified numerous molecular alterations in immune responses, inflammatory processes and metabolic pathways to be associated with the disease severity in COVID-19-adults, and the results were highly consistent with other following studies 22, 59-67. To compare the major findings of this study to other existing studies, the FC/NAV values of COVID-19-adults against healthy adults or non-COVID-19 patients for the 5 proteins and 5 metabolites were directly taken from the literature 54, 58, 60, 61, 63, 67, if available. From the results, it could be found that the 10 molecules were differentially expressed in COVID-19-children with a stronger extent than in COVID-adults (Table S16).

In our results, F11, F9, FGG, FGA, SERPINA5, SERPINC1, and SERPINF2 are involved in the coagulation cascade, and significantly higher expressed in COVID-19-children compared with COVID-19-adults (Figure S4C, Table S6). The coagulation system plays important roles in immune responses against infections, prevents damage to tissues, and facilitates the repair of damaged areas 68. However, over-activation of the coagulation cascade during the immune response to infection usually exacerbates production of pro-inflammatory cytokines, and coagulation-induced thrombin also exerts the activity to further augment the inflammation 68. Therefore, our findings suggested that COVID-19-associated coagulation and the accompanying immune response/inflammatory processes in COVID-19-children are strongly induced. On the other hand, an inflammation-associated protein, ENO1, was down-regulated in COVID-19-children against COVID-19-adults, indicating that anti-inflammatory processes were also actively triggered in the proteomic level. Also, the levels of many negative regulators of inflammation and oxidation, such as TRP, IAAld, DHOA, mannitol, MMA and UA in COVID-19-children were significantly up-regulated compared with those COVID-19-adults, supporting the strong antagonistic effect. Moreover, our findings showed that these metabolites not only relieved the expressions of various pro-inflammatory factors, but also exhibited an unexpected activity to inhibit MHV replication in cells. These results further supported that the immune response was strengthened in COVID-19-children, and suggested that SARS-CoV-2 replication in COVID-19-children was restricted by the enhanced levels of COVID-19-associated plasma molecules. Thus, we speculated that the immune system of COVID-19-children might be in a relatively balanced state, in which its activation is stronger than that of COVID-19-adults, and is sufficient to restrict SARS-CoV-2 infection and the collateral damages.

Identification of the alternations of plasma molecules in COVID-19-children against in healthy children or COVID-19-adults also provided promising therapeutic agents for COVID-19. Here, we tested the effects of MMA, DHOA, TRP and mannitol on the expression levels of multiple pro-inflammatory cytokines, as well as the viral replication in MHV-infected cells. Interestingly, the changes in the types of cytokines were different in response to distinct metabolite treatments, suggesting that the action to mode of these metabolites are dependent on different cellular signaling pathways. Moreover, we found that MMA or mannitol treatment can efficiently inhibit MHV replication. Mannitol is reported to be a hydroxyl radical scavenger that plays important roles in relieving inflammation 37. A recent study showed that the SARS-CoV-2 infection in monocytes triggers mitochondrial reactive oxygen species (ROS) production, which induces the stabilization of hypoxia-inducible factor-1α (HIF-1α) and consequently metabolism reprogramming that facilitates the viral replication and inhibits immune responses 69. It is possible that the hydroxyl radical scavenging activity of mannitol relieves the cellular level of ROS, and in turn suppresses MHV replication. For MMA, its exact role in regulation of immune response or inflammation is not clear. It was showed that MMA treatment in A549 cells triggered the induction of SOX4 38. Interestingly, a recent study found that SOX4 could suppress hepatitis B virus replication via inhibiting hepatocyte nuclear factor 4α (HNF4α) 70. Moreover, our findings showed that IAAld and uric acid (UA) were also significantly up-regulated in COVID-19-children compared with those in COVID-19-adults. However, there are many gaps in their relationships with viral replication and inflammation. The detailed roles of the identified metabolites specifically altered in COVID-19-children need to be further investigated, and the clinical usage of these therapeutic metabolites remains to be tested.

### Limitations of the study

There are a number of limitations in our study. First, the plasma samples of COVID-19-children were collected at the early stage of the COVID-19 outbreak in China. At that time, the number of hospitalized COVID-19-children was limited. Thus, the sample size in this study was relatively small. Another possible drawback of this work is that the intrinsic differences between children and adults might not be fully excluded, although we used NAVs of proteins and metabolites for the comparison of COVID-19-children and COVID-19-adults, after normalization using the multi-omic data of healthy children and healthy adults, respectively. Third, two optimal biomolecular combinations were computationally prioritized by iBM, in which total RMSE values were calculated to estimate and reduce the prediction bias. However, over-fitting might not be fully avoided for the finally determined models. Moreover, it was demonstrated that proteomics-based findings were highly variable across different studies 54, 58, 59. Many factors, such as the cohorts enrolled from different countries or regions, the differences in age, sex, body mass index (BMI), physical conditions and other characteristics of the enrolled patients, including different MOI stages, different procedures of sample preparation and different types of data analysis platforms, might considerably influence the final results 71. Thus, although experiments were conducted to validate the omics-based predictions in this study, we anticipated that the enrollment of more plasma samples of COVID-19-children, probably from multiple centers, would be helpful to further validate our findings. Finally, the roles of more proteins and metabolites specifically altered in COVID-19-children need to be further investigated, and whether the prioritized metabolites could be taken as clinically therapeutic agents also remained to be dissected.

In summary, our findings provided a highly valuable multi-omic data resource for the research community to better understand COVID-19-associated host responses. We identified a number of proteins or metabolites specifically altered in COVID-19-children, shed lights on the pathogenesis of COVID-19 in children, and provided potential therapeutic agents for treatment of the disease.

## Materials and Methods

### Ethics and Human Subjects

All work performed in this study was approved by the Guangzhou Women and Children's Medical Center Ethics Committee. Written informed consent was waived by the Ethics Commission of the designated hospital for emerging infectious diseases. Diagnosis of SARS-CoV-2 infection was based on the New Coronavirus Pneumonia Prevention and Control Program (6^th^ edition) published by the National Health Commission of China 25. Nasopharyngeal swabs were collected upon admission and then every 1-3 d throughout the hospitalization period. SARS-CoV-2 was tested through real-time RT-PCR of 2019-nCoVRNA as previously reported 6, 72. Healthy children were recruited at Guangzhou Women and Children's Medical Center. The throat swab tests and serological tests of healthy children were negative for SARS-CoV-2. All blood samples were collected after fasting overnight, and then added with ethylene diamine tetraacetic acid (EDTA) plus potassium (K^+^). All the blood samples were treated according to the biocontainment procedures of the processing of SARS-CoV-2-positive samples.

### Preparation of protein and peptide samples

For each serum sample, the cellular debris was removed by centrifugation at 12,000 g at 4 °C for 10 min. Then, the supernatant was transferred to new centrifuge tubes. The top 12 high abundance proteins were removed by Pierce™ Top 12 Abundant Protein Depletion Spin Columns Kit (Thermo Fisher). Finally, the protein concentration was determined with BCA kit (Beyotime Biotechnology) according to the manufacturer's instructions.

For digestion, the protein solution was reduced with 5 mM dithiothreitol (Sigma-Aldrich) for 30 min at 56 °C and alkylated with 11 mM iodoacetamide (Sigma-Aldrich) for 15 min at room temperature in darkness. The protein sample was made using buffer exchange by 8 M urea (Sigma-Aldrich) three times, and then using buffer exchange by the label buffer three times. Finally, trypsin (Promega) was added at 1:50 trypsin-to-protein mass ratio for the digestion overnight at 37 °C. The peptides were recovered by centrifugation at 12,000 g at room temperature for 10 min, and the recovery step was repeated by H_2_O.

For TMT labeling, the 30 samples were equally divided into two groups according to the comparison design and processed according to the manufacturer's protocol for TMTpro^TM^ 16plex Label Reagent (Thermo Fisher) kit (Table S1). For each batch, a pooling mixture of all the 30 plasma samples was included and labeled as a standard control to eliminate the batch effect. Then, one unit of TMTpro reagent (defined as the amount of reagent required to label 100 μg of proteins) were thawed and reconstituted in 10 μL acetonitrile (ACN, Thermo Fisher). The peptide mixtures were then incubated for 2 h at room temperature and pooled, desalted and dried by vacuum centrifugation. The labeling efficiency (calculated from the ratio of number of TMT labeled sites divided by number of all the potential labeling sites) had to pass the threshold of 95% before proceeding to the fractionation step.

The sample was then fractionated into fractions by high pH reverse-phase high-performance liquid chromatography (HPLC) using Agilent 300Extend C18 column (5 μm particles, 4.6 mm ID, 250 mm length). Briefly, peptides were first separated with a gradient of 2% to 32% ACN in 10 mM ammonium bicarbonate pH 10 over 60 min into 60 fractions 73. Then, the peptides were combined into 6 fractions and dried by vacuum centrifuging.

### LC-MS/MS-based proteomic analysis

LC-MS/MS data acquisition was carried out on an Exploris 480 mass spectrometer coupled with an Easy-nLC 1200 system (both Thermo Scientific) 55, 74. Peptides were loaded onto a home-made reversed-phase analytical column (100 μm × 250 mm, 1.9 μm particle size, 120 Å pore size, Dr. Maish GmbH, Germany), and then separated. Mobile phase A (2% ACN, 0.1% formic acid [FA]) and mobile phase B (90% ACN, 0.1% FA) were used to establish a 60 min separation gradient (0 min - 7% B; 4 min - 11% B; 53 min - 32% B; 57 min - 80% B; 60 min - 80% B). A constant flow rate was set at 500 nL/min. For the analysis in data-dependent acquisition (DDA) mode, each scan cycle consisted of one full-scan mass spectrum (R = 60 K, AGC = 100%, max IT = 50 ms, scan range = 400-1200 m/z) followed by 25 MS/MS events (R = 45 K, AGC = 100%, max IT = Auto). High energy collision dissociation (HCD) collision energy was set to 35. Isolation window for precursor selection was set to 1.6 Da. Former target ion exclusion was set for 30 s.

### Protein database search

MS/MS raw data were analyzed with Proteome Discoverer (v2.4.1.15) using the Andromeda database search algorithm (Thermo Fisher). The reference database contained 20,380 Swiss-Prot/reviewed human protein sequences downloaded from the UniProt database (https://www.uniprot.org/proteomes/UP000005640, on November 15, 2019), and reverse decoy sequences were generated. Then, spectra files were searched against the merged database using the following parameters: Type, TMT; Variable modifications, Oxidation (M), Acetyl (Protein N-term); Fixed modifications, Carbamidomethyl (C), TMTpro (peptide N-Terminus), TMTpro (K); Digestion, Trypsin (Full). The MS1 match tolerance was set as 10 parts per million (ppm); the MS2 tolerance was set as 0.02 Da. Search results were filtered with 1% false discovery rate (FDR) at both protein and peptide levels. Proteins denoted as decoy hits, or only identified by sites were removed, and the remaining proteins were used for further analysis.

### Extraction of hydrophilic and hydrophobic metabolites

To extract hydrophilic compounds, each sample was thawed on ice. Then, 6 volumes of ice-cold methanol (Merck) was added to 1 volume of plasma. The mixture was whirled for 3 min and then centrifuged at 12,000 g at 4 °C for 5 min. The supernatant was collected and stored at - 20 °C. After 30 min, the sample was centrifuged at 12000 g at 4 °C for 3 min, and the supernatant was collected and subjected to LC-MS/MS analysis.

To extract hydrophobic compounds, each sample was thawed on ice, whirled around 10 s, and then centrifuged with 3000 g at 4 °C for 5 min. Then, 250 μL methanol, 750 μL methyl tertiary-butyl ether (MTBE, Merck), and 13 internal standard mixtures (Sigma-Aldrich) were added to 50 μL of one sample. The mixture was homogenized and whirled for 2 min, added with 200 μL of water, whirled for 1 min, and then centrifuged with 12,000 g at 4 °C for 10 min. The 200 μL supernatant was extracted and concentrated. The powder was re-dissolved with 200 μL mobile phase B (90% ACN, 0.1% FA), and then and subjected to LC-MS/MS analysis.

### LC analysis of hydrophilic and hydrophobic compounds

For quality control of the metabolomic analysis, we pipetted 10 μL of each of the 30 plasma samples to pool a mixture, which was equally separated into 4 parts. When running sample sets on the UPLC column, 1 part of the control sample was first added, and the remaining 3 parts were sequentially injected after per 10 samples.

The sample extracts of hydrophilic compounds were analyzed using the ultra-performance liquid chromatography (UPLC) of a LC-MS/MS system (Shim-pack UFLC SHIMADZU CBM A system, MS, QTRAP® 6500+ System). The samples were injected onto a Waters HSS T3 column (1.8 µm, 2.1 mm × 100 mm). The column temperature, flow rate and injection volume were set 40 °C, 0.4 mL/min and 2 μL, respectively. Mobile phase A (2% ACN, 0.1% FA) and mobile phase B (90% ACN, 0.1% FA) were used to establish a 12 min separation gradient (0 min - 5% B; 11 min - 90% B; 12 min - 5% B).

The sample extracts of hydrophobic compounds were also analyzed using the same UPLC (Shim-pack UFLC SHIMADZU CBM A system, MS, QTRAP® 6500+ System). The samples were injected onto a Thermo Accucore™ C30 column (2.6 μm, 2.1 mm × 100 mm). The column temperature, flow rate and injection volume were set 45 °C, 0.35 mL/min and 2 μL, respectively. Mobile phase A (2% ACN, 0.1% FA) and mobile phase B (90% ACN, 0.1% FA) were used to establish a 20 min separation gradient (0 min - 20% B; 2 min - 30% B; 4 min - 60% B; 9 min - 85% B; 14 min - 90% B; 15.5 min - 95% B; 17.3 min - 90% B; 20 min - 20% B).

### MS/MS-based analysis of hydrophilic and hydrophobic compounds

Triple quadrupole (QQQ) scans with linear ion trap (LIT) were acquired using the LC-MS/MS system (Shim-pack UFLC SHIMADZU CBM A system, MS, QTRAP® 6500+ System). This system was equipped with an electrospray ionization (ESI) Turbo Ion-Spray interface, which could be operated in positive and negative ion modes and controlled by Analyst 1.6.3 software package (Sciex). The parameters of the ESI source operation were set as: ion source, turbo spray; source temperature, 550 °C; ion spray voltage, 5500 V in the positive ion mode or -4500 V in the negative ion mode; collision gas, 5 psi; ion source gas I, 45 psi; ion source gas II, 55 psi; curtain gas, 35 psi. The quantification of metabolites was accomplished using targeted multiple reaction monitoring (MRM) approach 75. Instrument tuning and mass calibration were performed with 10 and 100 μM polypropylene glycol solutions in QQQ and LIT modes, respectively. Declustering potential and collision energy for individual MRM transitions was done with a further optimization. A specific set of MRM transitions were monitored for each period according to the metabolites within this period. The analysis of each sample was conducted by both positive and negative ion modes, and the MRM transitions were listed in Table S17.

### Data analysis of plasma hydrophilic and hydrophobic metabolites

The MS/MS data were processed by Analyst 1.6.3 software package (Sciex). The reproducibility of metabolite extraction and detection were judged by total ion current and multiple peaks of MRM quantifications. According to the retention time and mass-to-charge ratio, the identification of both hydrophilic and hydrophobic metabolites was performed using a home-made metadata database and other existing metabolomic databases, including MassBank (http://www.massbank.jp/) 76, KNApSAcK (http://www.knapsackfamily.com) 77, HMDB (http://www.hmdb.ca/) 78, and METLIN (http://metlin.scripps.edu/index.php) 79 (Table S3).

For quantification of plasma metabolites, the metabolomic data was analyzed using MultiQuant software package 3.0.2 (Sciex), which automatically integrated and calibrated the chromatographic peaks. The IBA of each metabolite was calculated by the peak area of each chromatographic peak. To ensure the data quality, we calculated coefficient of variation (CV) values of all metabolites, and removed low-quality hits whose CV values were larger than 0.5.

### Reuse of the proteomic and metabolomic data of adults

Previously, we prepared two cohorts for the plasma proteomic profiling of COVID-19-adults and healthy adults 23, 24. The first cohort contained 5 patients with fatal outcome, 7 patients diagnosed as severe symptoms, 10 patients diagnosed as mild symptoms, and 8 healthy volunteers. The second cohort contained 9 patients with fatal outcome, 6 patients diagnosed as severe symptoms, 6 patients diagnosed as mild symptoms, and 5 healthy volunteers. For patients with multiple blood samples taken at different time points, only the sample collected at the first time after hospitalization was considered in this study. In total, the proteomic data set was derived from 56 plasma samples of 43 COVID-19-adults and 13 healthy adults.

For the metabolomic profiling, the cohort contained 34 COVID-19-adults including 9 patients with fatal outcome, 11 patients diagnosed as severe symptoms, and 14 patients diagnosed as mild symptoms, as well as 10 healthy volunteers 23, 24. Again, for patients with multiple samples taken at different time points, only the sample collected at the first time after hospitalization was considered.

### Data normalization and imputation

For each batch of the plasma proteomic data, the IBA of a protein in one sample was first normalized using its corresponding expression in the control of the same batch to calculate the RPA, which eliminated the batch effect prior to the comparative analysis of the samples of COVID-19-children and healthy children.

To identify molecular alterations exclusively in COVID-19-children *CC* but not healthy children *HC* or COVID-19-adults *AC* samples, the mean RPA value of a protein *i* in* HC* or healthy adults *HA* was first calculated as below:


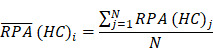



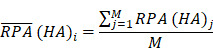


Where* N* and *M* denoted the numbers of *HC* and *HA* cases, respectively. Then, the NAV of the protein* i* in *CC* or *AC* samples was calculated as below:


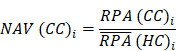



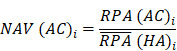


Analogously, the NAVs of all metabolites in each *CC* or *AC* sample were also computed.

To ensure the data quality for identification of potential DEPs or DEMs, we only reserved proteins or metabolites quantified in > 80% samples (575 proteins or 1155 metabolites in > 24 samples for the multi-omic analysis of COVID-19-children *vs*. healthy children, 332 proteins in > 68 samples for the proteomic analysis of COVID-19-children *vs*. COVID-19-adults, and 783 metabolites in > 59 samples for the metabolomic analysis of COVID-19-children *vs*. COVID-19-adults). Using the normal distribution imputation, the missing values were imputed with values representing a normal distribution around the detection limit of the mass spectrometer. For each sample, the mean and standard deviation (S.D.) of the distribution of the raw protein or metabolite intensities were calculated. Then a new distribution with a downshift of 1.8 S.D. and a width of 0.3 S.D. was automatically modeled. The total data set was imputed before statistical analysis. After imputation, the mean *μ* and S.D *σ* were counted for each protein or metabolite in COVID-19-children and healthy children, respectively, and CV was calculated as below:


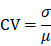


Before model training, the proteomic or metabolomic data of each sample was further normalized using the z-score transformation, one of the mostly used normalization methods. For each sample, the median expression value *m* and S.D. *δ* were first calculated for the proteomic or metabolomic data. For a protein or metabolite *i* with the abundance of NAV_i_, its normalized *z*-score was calculated as below:


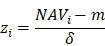


After transformation, the *z*-scores of proteins or metabolites followed a logarithmic normal distribution (log_2_) centered at zero.

The proteomic and metabolomic data normalization and imputation were conducted using Perseus 1.6.14 80. To test whether different types of patients could be distinguished, PCA was performed using Scikit-learn 0.22.1 (https://scikit-learn.org/stable/), a useful toolkit for data mining and analysis. The Pearson correlation analysis was performed by an R packge, corrplot (https://cran.r-project.org/web/packages/corrplot/index.html).

### Statistical analysis of the quantitative omic data

Using RPA values of the proteomic data and IBA values of the metabolomic data, we identified potential DEPs and DEMs that were significantly altered in COVID-19-children against healthy children. Then, using NAVs of the omic data, we further identified potential DEPs and DEMs that were significantly altered in COVID-19-children against COVID-19-adults. The FC value was calculated based on the mean of the same patient group for each pair of groups, and proteins or metabolites with |log_2_(FC)| > 0.25 were reserved. The statistical significance was calculated for reserved proteins and metabolites, using the unpaired two-sided Welch's t-test and adjusted *P* values were calculated using Benjamini & Hochberg correction (Adjusted *P* < 0.05). The statistical analyses were conducted using the ttest_ind function in scipy.stats.

### The enrichment analysis

The two-sided hypergeometric test was adopted for the GO- or KEGG-based enrichment analysis of the DEPs or DEMs. Here, we defined:

*N* = number of human proteins or metabolites annotated by at least one term

*n* = number of human proteins or metabolites annotated by term *t*

*M* = number of the DEPs or DEMs annotated by at least one term

*m* = number of the DEPs or DEMs annotated by term *t*

Then, the E-ratio was calculated, and the *P* value was computed with the hypergeometric distribution as below:

E-ratio = 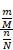


P value = 
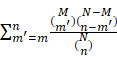
, (E-ratio > 1)

In this study, adjusted *P* values were calculated using Benjamini & Hochberg correction and only statistically over-represented GO terms for the proteomic data and KEGG pathways for the metabolomic data were considered. GO annotation files (released on 03 January 2020) were downloaded from the Gene Ontology Consortium Web site (http://www.geneontology.org/), and in total we obtained 19,288 human proteins annotated with at least one GO biological process term. KEGG annotation files (released on 4 September 2020) were downloaded from the ftp server of KEGG (ftp://ftp.bioinformatics.jp/), which contained 6,182 metabolites annotated with at least one KEGG pathway term. Proteins have both GO and KEGG annotations, whereas GO annotations were more integrative. Metabolites only have KEGG annotations. Thus, GO- and KEGG-based enrichment analyses were separately conducted for DEPs and DEMs.

### Performance evaluation

To evaluate the accuracy of iBM, the numbers of true positive (*TP*), true negative (*TN*), false positive (*FP*) and false negative (*FN*) hits were counted. Then, we calculated two measurements, including sensitivity (*Sn*), specificity (*Sp*) as below:


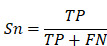



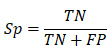


The 5-fold cross-validation was performed, while *Sn* and *Sp* values were calculated, respectively. The receiver operating characteristic (ROC) curve was illustrated and AUC value was calculated based on *Sn* and 1-*Sp* scores.

To estimate the prediction bias of a model, the root mean squared error (RMSE), an important measure of the residuals between predicted values and observed values, was calculated as below:


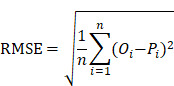


Where *n* represented the number of plasma samples in the data set. *P_i_* denoted the predicted probability value ranged from 0 to 1, while *O_i_* was equal to 0 for non-COVID-19 cases and 1 for COVID-19 cases, respectively.

### Inference of optimal biomolecular combinations

We separately identified the optimal combination exclusively altered in COVID-19-children with a minimal RMSE for the proteomic and metabolomic data, by developing a three-step pipeline named iBM that included MDS, CCG, and FCP.

In the step of MDS, mutually identified DEPs or DEMs in COVID-19-children against healthy children and COVID-19-children against COVID-19-adults were reserved as a candidate pool. Then, CCG was adopted to select different sets of combinations with ≤ 5 proteins or metabolites. This number was much smaller than the sample size and could efficiently avoid over-fitting. From the pool, 10,000 candidate combinations were randomly generated for the proteomic and metabolomic data, respectively. The initial weight value of each protein or metabolite was set to 1.

In the step of FCP, the 5-fold cross-validation was conducted for model training. For each candidate combination, we randomly generated a training data set and a testing data set with a ratio of approximately 4:1. The testing data set was only used to test the performance but not for training, and the final total AUC value was calculated as below:


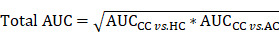


The least absolute shrinkage and selection operator (LASSO, L1 regularization) penalty and the ridge regression (L2 regularization) penalty in PLR 26-28, were iteratively used to optimize the weight values of the 5 proteins or metabolites. To simplify the combination, one or multiple proteins or metabolites were randomly dropped if the total AUC value of the 5-fold cross-validation was increased. Such a procedure was repeatedly performed until the AUC value was not increased any longer. All combinations with a total AUC equal to 1 were reserved for the proteomic and metabolomic data, respectively (Table S12-S13). The total RMSE value of all samples was calculated for each combination, and the final result was determined based on the minimal total RMSE value.

The PLR algorithm was implemented in Python 3.7 with Scikit-learn 0.22.1. The source code of iBM is available at: https://github.com/Ning-310/iBM.

### Cell culture and virus infection

The L2 cell line was kindly provided by Prof. Chen (Wuhan University, China) and maintained in Dulbecco's modified Eagle's medium (DMEM, Gibco) supplemented with 10% fetal bovine serum (Gibco), 100 U/mL penicillin and 100 μg/mL streptomycin at 37 °C in a humidified atmosphere with 5% CO_2_. The MHV strain A59 was kindly provided by Prof. Chen (Wuhan University, China).

MMA (STBF5304V), DHOA (SLCD3296) and mannitol (WXBD1141V) were commercially purchased from Sigma-Aldrich. TRP (10211562) was commercially purchased from Alfa Aesar. The CCK-8 assay was used to evaluate the cytotoxicity of MMA, DHOA, TRP and mannitol in L2 cells. Briefly, the cells were seeded into 96-well plates and incubated with increased concentrations from 0 μM to 1280 μM. After incubation at 37 °C for 12 h, the cell supernatant was replaced with fresh DMEM. Then, after incubation for additional 12 h, the CCK-8 solution was added for detection of the absorbance at 450 nm using a microplate reader (Infinite M200PRO). The CCK-8 kit was purchased from Dojindo.

For detection of the effects of MMA, DHOA, TRP and mannitol upon MHV infection, each one of the tested compounds at the concentration of 5 or 10 μM was added to L2 cells (4×10^5^ cells in each of the 12 wells in a cell culture-treated plate). After 1 h incubation, L2 cells were infected with MHV at MOI = 0.1. At 12 h post-infection, the infected L2 cells were collected and total cellular RNAs were extracted using Total RNA kit (Foregene). The viral RNA accumulation and the mRNA levels of IL-6, IL-1β, TNF-α, TGF-β and IL-10 were determined via qRT-PCR.

The qRT-PCR was performed with the primers specific for F9, F11, FGA, FGG, ENO1, IL-6, IL-1β, TNF-α, TGF-β and IL-10. The qRT-PCR was performed using One Step SYBR® PrimeScript™ PLUS RT-PCR Kit (Yeasen). All the primers used here were listed in Table S15.

## Supplementary Material

Supplementary figures.

Supplementary tables.

## Figures and Tables

**Table 1:** Clinical characteristics of COVID-19-children and healthy children enrolled in this study. *a*. ND, not detected.

	COVID-19-children (*n* = 18)	Healthy children (*n* = 12)
Age-year		
Median (IQR)	7 (5, 12)	6 (3, 7)
Sex-no. (%)		
Female	5 (27.78%)	4 (33.33%)
Male	13 (72.22%)	8 (66.67%)
Throat swab for SARS-CoV-2 (days)		
Median (IQR)	6 (4, 14)	0
Sampling time from the disease onset (days)		
Median (IQR)	7 (10, 13)	0
Co-infection-no. (%)		
Other viruses	ND*^a^*	ND
Bacteria	ND	ND
Fungus	ND	ND
Clinical outcome-no. (%)		
Discharged	18 (100%)	ND
Blood routine- Median (IQR)		
WBC (×10^9^/L, normal range 5-12)	6.90 (5.50, 8.55)	6.80 (6.53, 7.35)
Lymphocyte (×10^9^/L, normal range 1.5-4.8)	2.60 (2.07, 4.19)	3.38 (2.95, 3.94)
Neutrophil (×10^9^/L, normal range 2-7.2)	2.58 (2.15, 3.24)	2.74 (2.38, 3.38)
CD19+ (cells/μL, normal range 90-660)	396.35 (236.82, 800.53)	ND
CD3+ (cells/μL, normal range 690-2540)	1232.74 (967.60, 2305.85)	ND
CD3+CD4+ (cells/μL, normal range 410-1590)	655.44 (459.88, 1150.56)	ND
CD3+CD8+ (cells/μL, normal range 190-1140)	482.67 (386.66, 859.49)	ND
NK (cells/μL, normal range 90-590)	334.34 (194.04, 438.04)	ND
Platelet (×10^9^/L, normal range 140-440)	299.50 (263.00, 333.75)	307.00 (242.50, 362.75)
RBC (×10^12^/L, normal range 4-4.5)	4.66 (4.37, 5.36)	4.79 (4.29, 4.93)
Haemoglobin (g/L, normal range 105-145)	124.50 (119.00, 141.75)	ND
APTT (s, normal range 28-45)	40.50 (37.40, 42.60)	ND
PT (s, normal range 11-15)	13.40 (13.15, 13.90)	ND
D-dimer (μg/mL, normal range, 0-1.5 )	0.31 (0.26, 0.40)	ND
Cytokines- Median (IQR)		
IFN-γ (pg/mL, normal range 0-6.56)	4.62 (3.97, 6.07)	ND
IL-10 (pg/mL, normal range 0-8.14)	1.98 (1.74, 2.91)	ND
IL-12p70 (pg/mL, normal range 0-6.9)	2.89 (1.77, 4.38)	ND
IL-17A (pg/mL, normal range 0-3.71)	5.98 (3.67, 8.76)	ND
IL-1β (pg/mL, normal range 0-3.12)	0.94 (0.59, 1.70)	ND
IL-2 (pg/mL, normal range 0-5.03)	4.33 (2.71, 5.01)	ND
IL-22 (pg/mL, normal range 0-2.61)	2.53 (1.66, 4.40)	ND
IL-4 (pg/mL, normal range 0-4.62)	3.37 (2.89, 5.42)	ND
IL-5 (pg/mL, normal range 0-3.73)	2.45 (1.94, 3.47)	ND
IL-6 (pg/mL, normal range 0-8.88)	3.84 (2.33, 6.58)	ND
IL-8 (pg/mL, normal range 0-15.71)	6.55 (4.37, 9.34)	ND
TNF-α (pg/mL, normal range 0-5.35)	5.35 (4.12, 6.95)	ND
