# Supplementary material for: Multi-omic profiling of plasma reveals molecular alterations in children with COVID-19
Source: Theranostics. 2021 Jul 6;11(16):8008–26. doi: 10.7150/thno.61832 (PMC8315065; doi:10.7150/thno.61832)
Supplement: Supplementary file 1 — Supplementary figures. [file thnov11p8008s1.pdf]

Supplementary Materials for

Multi-omic profiling of plasma reveals molecular alterations in children with COVID-19

Chong Wang[1,2†], Xufang Li[1†], Wanshan Ning[3†], Sitang Gong[1], Fengxia Yang[1], Chunxiao Fang[1], Yu Gong[1], Di Wu[2], Muhan Huang[2], Yujie Gou[3], Shanshan Fu[3], Yujie Ren[1,2], Ruyi Yang[2], Yang Qiu[2]*, Yu Xue[3]*, Yi Xu[1]*, Xi Zhou[1,2]*

[†]These authors contributed equally

*Corresponding author. Email: zhouxi@wh.iov.cn (X.Z.), xueyu@hust.edu.cn (Y.Xue), xuyi70@163.com (Y.Xu), and yangqiu@wh.iov.cn (Y.Q.)

This PDF file includes:

Figs. S1 to S6

Other Supplementary Materials for this manuscript include the following:

Datasheet Table S1 to S17

Supplementary results

The quality control and analysis of the multi-omic data

The proteomic profiling detected 9445 peptides from the 30 plasma samples of children, with an average number of 4716.1 peptides per sample (Fig. S1A). We mapped the peptides to corresponding protein sequences, and quantified proteins using the reporter ion MS2 module of the MaxQuant software package [1]. We found that 757 proteins were quantified in at least one sample (Table S3), with an average number of 666 proteins per sample (Fig. S1B). We checked the raw MS/MS data and found that 4877 peptides (48.4%) could be matched by ≥ 2 spectral counts (Fig. S1C). The average spectral counts were calculated as 2.6 for all peptides, indicating a high quality of peptide identification. Also, we found that 626 proteins (79.2%) could be traced and supported by ≥ 2 peptides, with an average number of 11.1 peptides (Fig. S1D). Thus, our proteomic data was also highly reliable at the protein level. For the metabolomic profiling, we added 4 equal parts of a control sample by pooling the 30 plasma samples, and the principal component analysis (PCA) demonstrated that the 4 parts of the control sample could be closely clustered, indicating a high quality of metabolomic quantification (Fig. S1E). In total, we obtained 1171 metabolites from the 30 samples (Table S4), with an average number of 1140.7 metabolites per sample (Fig. S1F).

From the multi-omic profiling, the intensity-based abundances (IBAs) of proteins and metabolites were obtained. For each batch of the proteomic data, the relative protein abundances (RPA) were obtained by normalizing the control sample, and proteins not quantified in control were discarded. To ensure the data quality, only 575 proteins and 1155 metabolites mutually quantified in > 80% samples for further analysis, and a heatmap was illustrated after a hierarchical clustering (Fig. S2). It could be found that the samples of COVID-19-children and healthy children had distinct molecular signatures. For each protein or metabolite, normal distribution imputation was applied to impute the missing values [2]. To measure the variability of proteomic and metabolomic quantification, the coefficient of variation (CV) was calculated for each molecule, with median values of 0.172 and 0.135 for the proteomic data, and 0.122 and 0.122 for the metabolomic data in the samples of COVID-19-children and healthy children, respectively (Fig. S3A). The low CV values supported a high reproducibility of the multi-omic quantification. Then, PCA was conducted for analyzing the proteomic (Fig. S3B) and metabolomic data (Fig. S3C). From the results, it was found that COVID-19-children and healthy children could be clearly

distinguished using either the data type, indicating biomolecules were significantly altered in both omic levels. In addition, we calculated the average Pearson correlation coefficient (PCC) for each pair of samples using the proteomic (Fig. S3D) or metabolomic data (Fig. S3E), and the results supported that similar molecular alterations in COVID-19-children.

References

1. Tyanova S, Temu T, Cox J. The MaxQuant computational platform for mass spectrometry-based shotgun proteomics. *Nat Protoc*. 2016; 11: 2301-19.
2. Tyanova S, Temu T, Sinitcyn P, Carlson A, Hein MY, Geiger T, et al. The Perseus computational platform for comprehensive analysis of (prote)omics data. *Nat Methods*. 2016; 13: 731-40.
3. Deng W, Wang Y, Liu Z, Cheng H, Xue Y. HemI: a toolkit for illustrating heatmaps. *PLoS One*. 2014; 9: e111988.

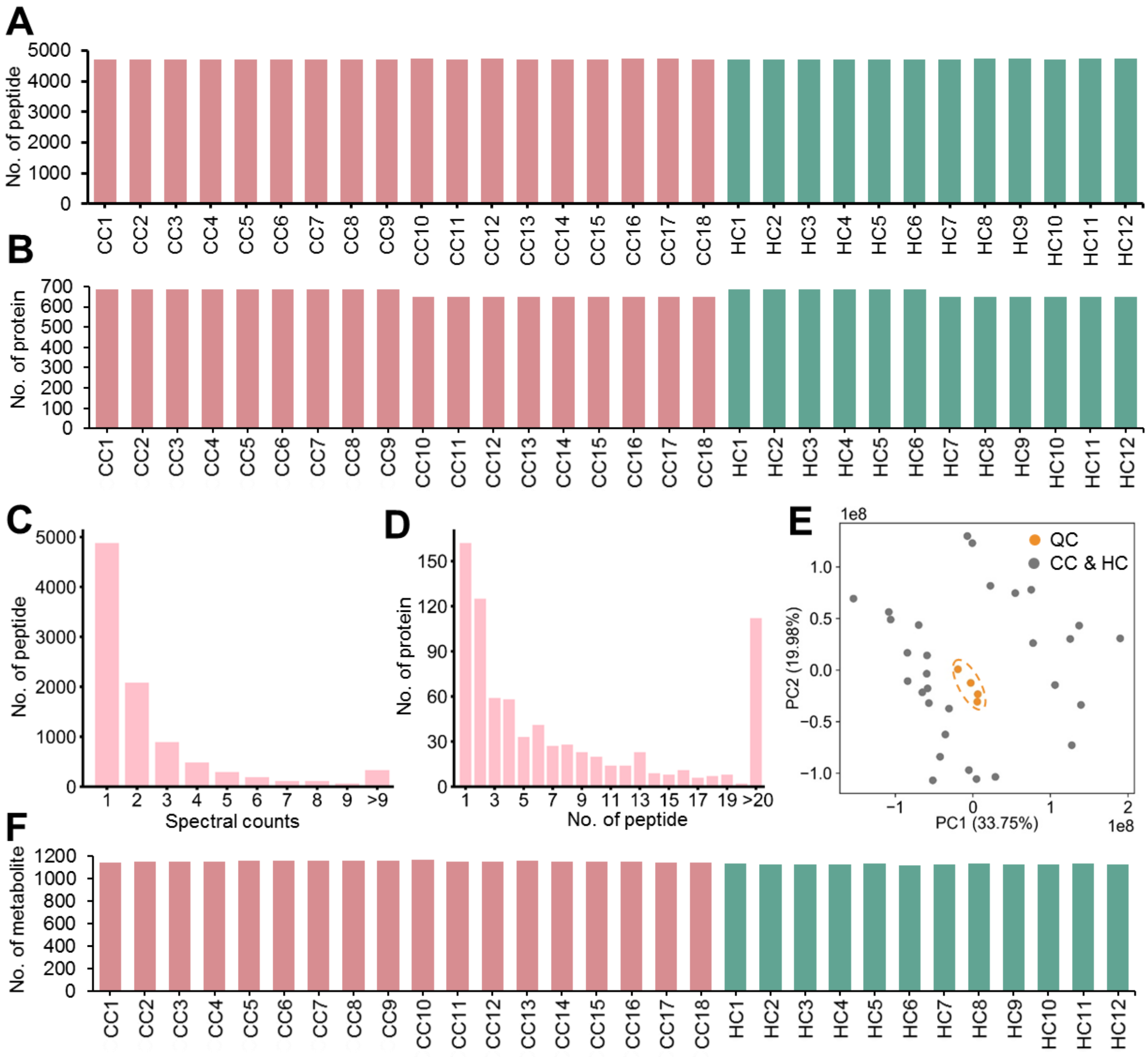

Figure S1. Proteomic and metabolomic profiling of plasma from the blood samples of COVID-19-children and healthy children. A,B The distribution of numbers of quantified (a) peptides and (b) proteins in the 30 plasma samples. **C** The distribution of MS/MS spectral counts of quantified peptides. **D** The distribution of peptide numbers of quantified proteins. **E** PCA analysis of the 30 metabolomic data and 4 quality controls (QCs). We pipette 10 μL of each sample to pool a QC sample. **F** The distribution of MS/MS spectral counts of quantified metabolites. CC, COVID-19-children; HC, healthy children.

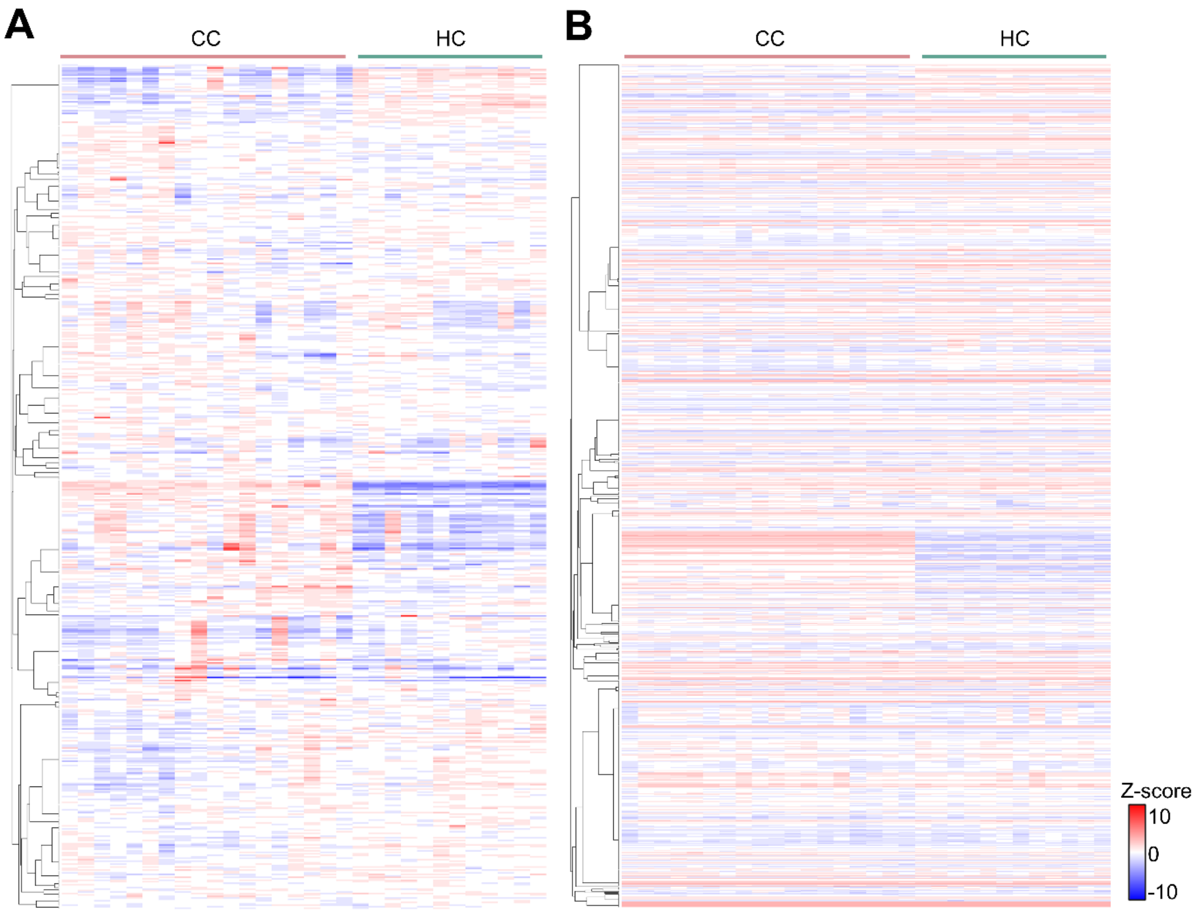

Figure S2. The heatmaps for the identified plasma proteins and metabolites in COVID-19-children and healthy children. A,B A hierarchical clustering was conducted for the 575 proteins (A) and 1155 metabolites (B) mutually quantified in > 80% samples, and the results were illustrated by a software package named Heatmap Illustrator (HemI) [3]. CC, COVID-19-children; HC, healthy children.

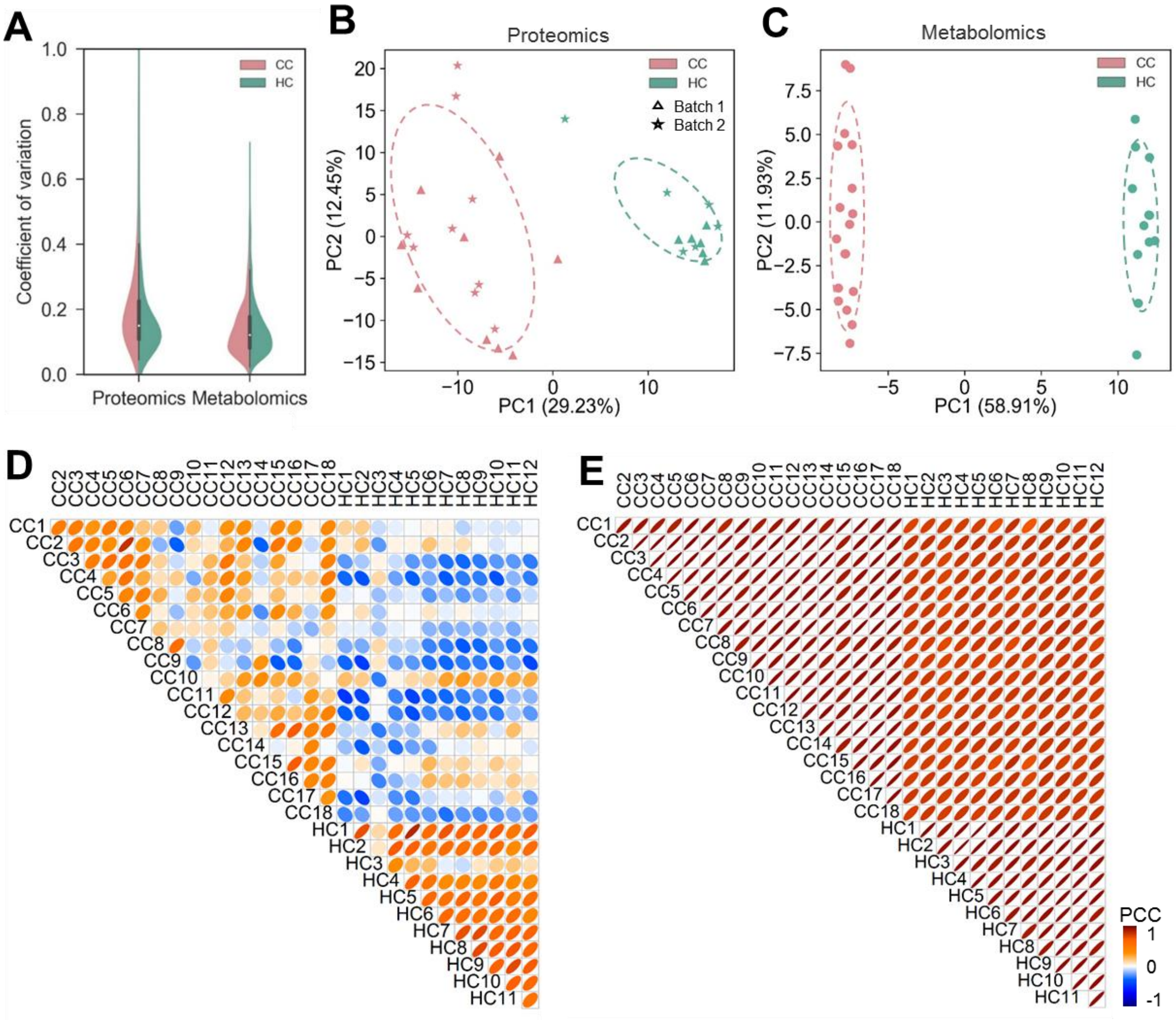

Figure S3. Quality control of proteomic and metabolomic data. A The distribution of CV values of the proteomic and metabolomic data. **B,C** The PCA results of the proteomic (B) and metabolomic (C) data. **D,E** PCC for each pair of samples using the proteomic (D) and metabolomic (E) data. CC, COVID-19-children; HC, healthy children; CV, coefficient of variation; PCC, pearson correlation coefficient; PCA, principal component analysis.

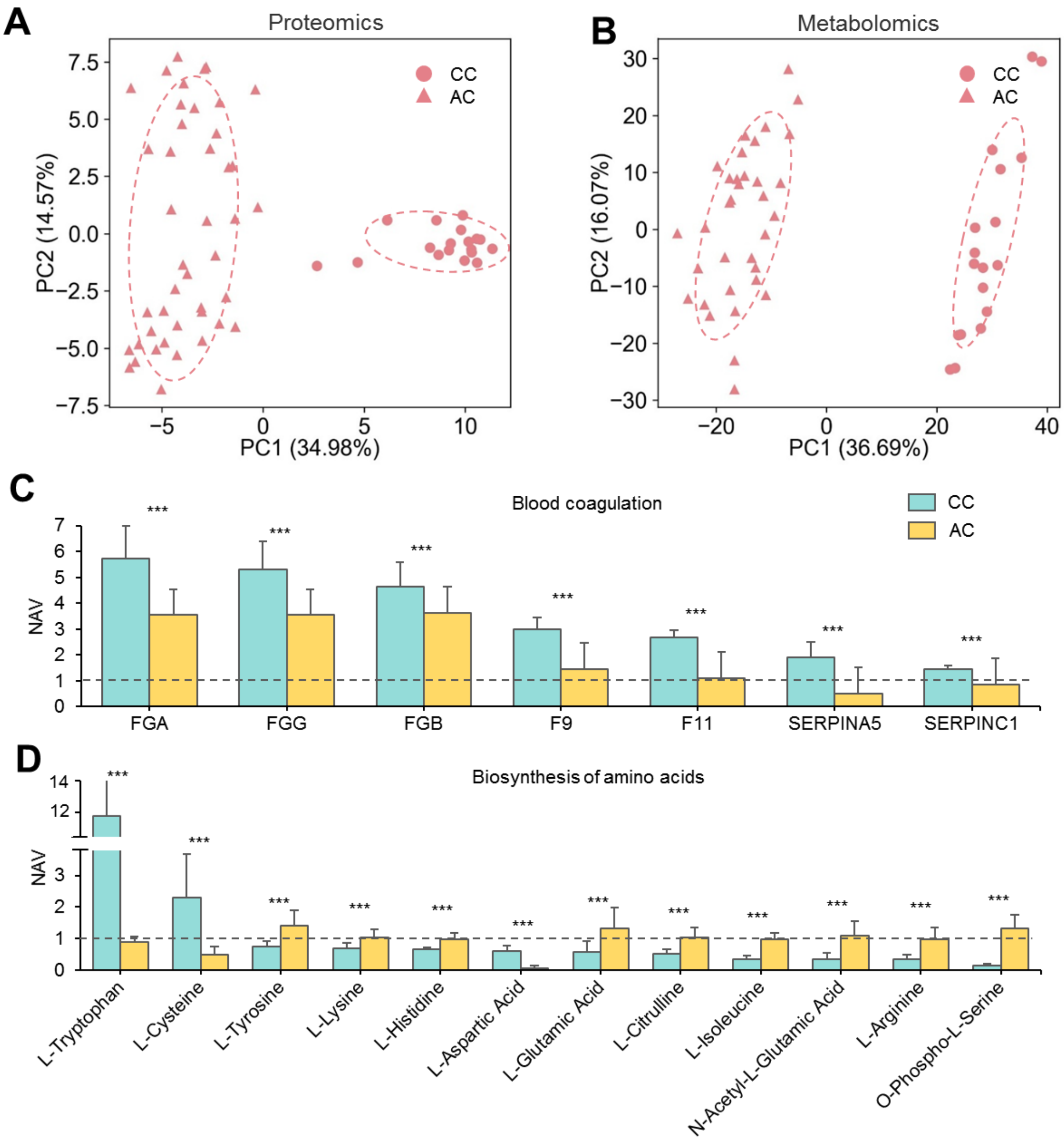

Figure S4. The alternations of molecules in COVID-19-children against COVID-19-adults, after normalization using the average expression values of these molecules in healthy children and healthy adults, respectively. A,B PCA analyses of the proteomic data of COVID-19-children and COVID-19-adults (A), and the metabolomics data of COVID-19-children and COVID-19-adults (B). **C** The abundance of proteins enriched in the process of blood coagulation in COVID-19-children and COVID-19-adults. **D** The abundance of metabolites enriched in the process of biosynthesis of amino acids in COVID-19-children and COVID-19-adults. Type or

paste caption here. Create a page break and paste in the Figure above the caption. CC, COVID-19-children; AC, COVID-19-adults; ***P < 0.001.

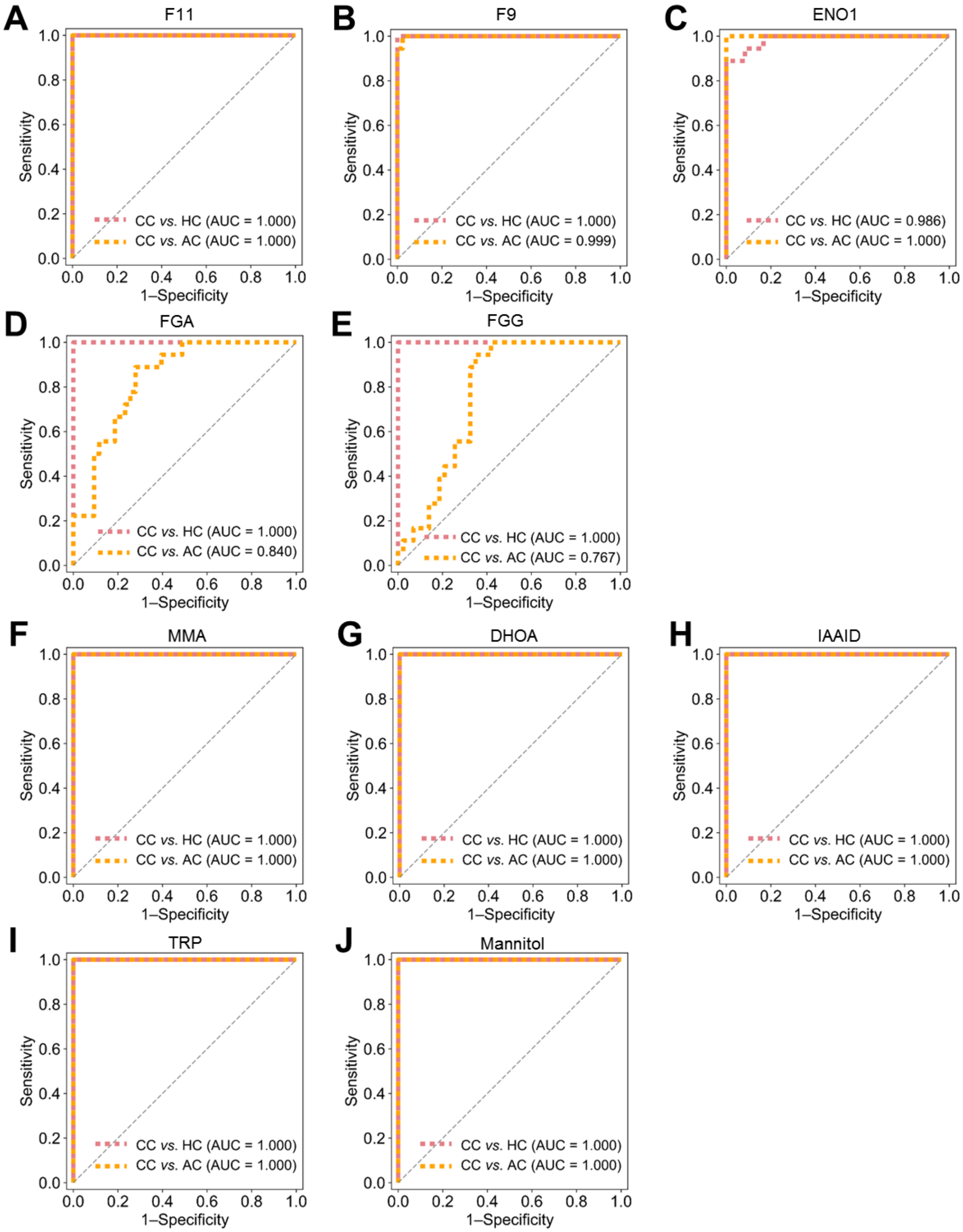

Figure S5. Individual biomarkers for classifying COVID-19-children from COVID-19-adults and healthy children. The receiver operating characteristic (ROC) curve and 5-fold cross-

validation area under curve (AUC) values of the indicated molecules to classifier the COVID-19-children from healthy children and COVID-19-adults. CC, COVID-19-children; HC, healthy children; AC, COVID-19-adults.

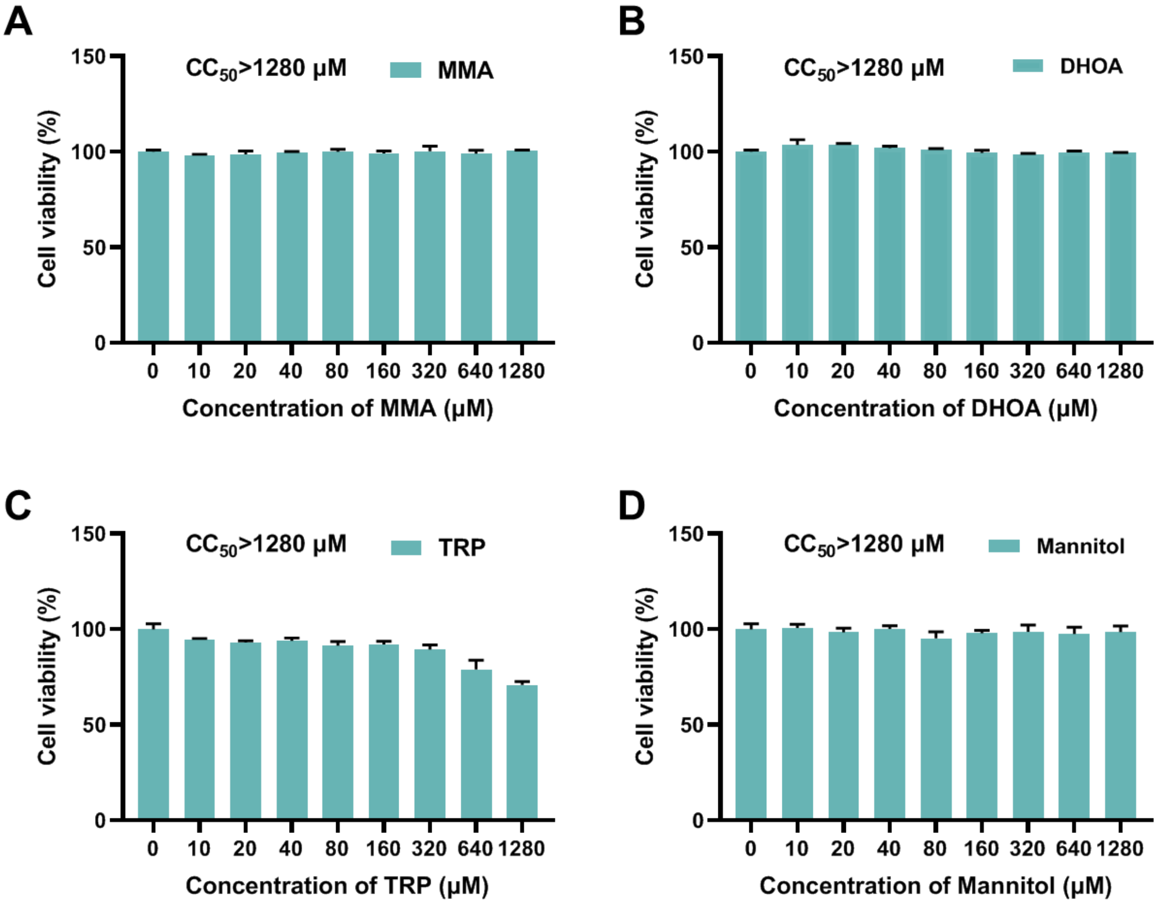

Figure S6. Cytotoxicity of metabolites MMA, DHOA, TRP and Mannitol in cell. Increasing concentrations of MMA, DHOA, TRP and Mannitol in DMEM with 2% FBS were added to L2 cells for 12 hr at 37 ℃. Cell viability of MMA (A), DHOA (B), TRP (C) and Mannitol (D) was determined by CCK-8 assay, and the absorbance at 450 nm was measured by microplate reader (Infinite M200PRO).
